# Design, synthesis, molecular docking studies and biological evaluation of thiazole carboxamide derivatives as COX inhibitors

**DOI:** 10.1186/s13065-023-00924-3

**Published:** 2023-03-06

**Authors:** Mohammed Hawash, Nidal Jaradat, Murad Abualhasan, Murat Kadır Şüküroğlu, Mohammed T. Qaoud, Deniz Cansen Kahraman, Heba Daraghmeh, Leen Maslamani, Mais Sawafta, Ala Ratrout, Linda Issa

**Affiliations:** 1 Department of Pharmacy, Faculty of Medicine and Health Sciences, An-Najah National University, Nablus, Palestine; 2 Department of Pharmaceutical Chemistry, Faculty of Pharmacy, Gazi University, Ankara, Turkey; 3 Cancer Systems Biology Laboratory, Graduate School of Informatics, Middle East Technical University, 06800 Ankara, Turkey

**Keywords:** Thiazole, NSAIDs, COX, HCT116, Molecular docking

## Abstract

**Background:**

Nonsteroidal anti-inflammatory drugs (NSAIDs) have been the most commonly used class of medications worldwide for the last three decades.

**Objectives:**

This study aimed to design and synthesize a novel series of methoxyphenyl thiazole carboxamide derivatives and evaluate their cyclooxygenase (COX) suppressant and cytotoxic properties.

**Methods:**

The synthesized compounds were characterized using ^1^H, ^13^C-NMR, IR, and HRMS spectrum analysis and were evaluated for their selectivity towards COX-1 and COX-2 using an in vitro COX inhibition assay kit. Besides, their cytotoxicity was evaluated using the Sulforhodamine B (SRB) assay. Moreover, molecular docking studies were conducted to identify the possible binding patterns of these compounds within both COX-1 and COX-2 isozymes, utilizing human X-ray crystal structures. The density functional theory (DFT) analysis was used to evaluate compound chemical reactivity, which was determined by calculating the frontier orbital energy of both HOMO and LUMO orbitals, as well as the HOMO–LUMO energy gap. Finally, the QiKProp module was used for ADME-T analysis.

**Results:**

The results revealed that all synthesized molecules have potent inhibitory activities against COX enzymes. The percentage of inhibitory activities at 5 µM concentration against the COX2 enzyme was in the range of 53.9–81.5%, while the percentage against the COX-1 enzyme was 14.7–74.8%. That means almost all of our compounds have selective inhibition activities against the COX-2 enzyme, and the most selective compound was **2f**, with selectivity ratio (SR) value of 3.67 at 5 µM concentration, which has a bulky group of trimethoxy on the phenyl ring that could not bind well with the COX-1 enzyme. Compound **2h** was the most potent, with an inhibitory activity percentage at 5 µM concentration of 81.5 and 58.2% against COX-2 and COX-1, respectively. The cytotoxicity of these compounds was evaluated against three cancer cell lines: Huh7, MCF-7, and HCT116, and negligible or very weak activities were observed for all of these compounds except compound **2f,** which showed moderate activities with IC_50_ values of 17.47 and 14.57 µM against Huh7 and HCT116 cancer cell lines, respectively. Analysis of the molecular docking suggests **2d**, **2e**, **2f**, and **2i** molecules were bound to COX-2 isozyme favorably over COX-1 enzyme, and their interaction behaviors within COX-1 and COX-2 isozymes were comparable to celecoxib, as an ideal selective COX-2 drug, which explained their high potency and COX-2 selectivity. The molecular docking scores and expected affinity using the MM-GBSA approach were consistent with the recorded biological activity. The calculated global reactivity descriptors, such as HOMO and LUMO energies and the HOMO–LUMO gaps, confirmed the key structural features required to achieve favorable binding interactions and thus improve affinity. The in silico ADME-T studies asserted the druggability of molecules and have the potential to become lead molecules in the drug discovery process.

**Conclusion:**

In general, the series of the synthesized compounds had a strong effect on both enzymes (COX-1 and COX-2) and the trimethoxy compound **2f** was more selective than the other compounds.

**Supplementary Information:**

The online version contains supplementary material available at 10.1186/s13065-023-00924-3.

## Background

Non-steroidal anti-inflammatory drugs (NSAIDs) are one of the most common analgesics that target cyclooxygenase (COX) iso-enzymes. They are used for different therapeutic applications worldwide. Due to their broad pharmacological activities, such as antipyretic, analgesic, and anti-inflammatory effects, they are considered one of the most appropriate families for treating various ailments, including rheumatism and arthritis, as well as their use as analgesics. Moreover, aspirin (acetylsalicylic acid), which belongs to this family, has been used for more than a 100 years [1, 2]. The biological synthesis of prostaglandin H2 from arachidonic acid (AA) is catalyzed by the COX enzyme [3, 4]. Prostaglandin H2 is the first intermediate in the process of synthesizing various prostacyclins, prostaglandins, and thromboxanes that have essential roles in many important pathological and physiological reactions [5]. COX-1 and COX-2 are the main isoforms of cyclooxygenase enzymes which are considered membrane-bounded enzymes [6]. The COX-1 enzyme is engaged in the production of many prostaglandins that are essential to preserving the functions of the gastrointestinal and cardiovascular systems [7], as well as the COX-2 enzyme is overexpressed in various pathophysiological conditions like inflammation [8].

Both COX-1 and COX-2 enzymes’ structures have similarities in the amino acids, and about 67% of these amino acids are identical, while the remaining amino acids are different, COX-1 has isoleucine (Ile523) instead of valine (Val523) in COX-2, and this difference makes the COX-2 binding pocket larger than the COX-1 binding pocket [9]. The long-term use of medications that mainly inhibit the COX-1 enzyme usually leads to GIT side effects such as ulcers, as well as may lead to kidney or liver damage [10], and because of that the researchers tried to develop selective NSAIDs such as valdecoxib, celecoxib, and rofecoxib to overcome the mentioned side effects [11]. However, the long-term use of these agents leads to a decrease in the biosynthesis of prostaglandin I2, which has developed cardiovascular side effects [12] and because of that, there is a necessity for safer and more selective inhibitors. Studies showed that tricyclic derivatives had better COX-2/COX-1 ratios compared to conventional NSAIDs such as aspirin and ketoprofen [13]. COX-2 enzyme is usually overexpressed in several sorts of human cancers, the biological studies consistently explained that COX-2 inhibitors compounds can inhibit the tumour progression and metastasis in several animal models of cancer. Essinaly, several observations have also shown COX-2 inhibitors can act synergistically with currently used anticancer agents [14], moreover some studies have suggested that COX inhibitors, particularly COX-2 inhibitors, may have anti-tumor effects and could be used as a potential treatment for cancer [15, 16].

**Fig. 1:**
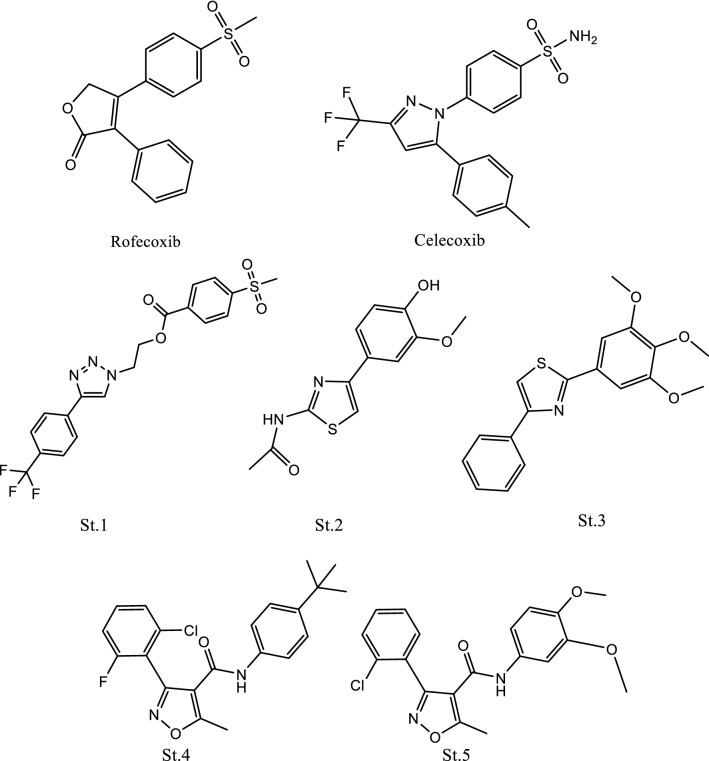
Chemical structures of COX inhibitors with heterocyclic rings including furanone (Rofecoxib), pyrazole (Celecoxib), thiazole (St.1-St.3), and Isoxazole (St.4 and St.5)

Rofecoxib and celecoxib (Fig. 1) are selective drugs that reached the market, and chemically they contain heterocyclic rings with COX inhibitory activity, as well as recently synthesized pyrazole or tetrazole derivatives showed selectivity and potent inhibitory activities on COX enzymes. Compound St.1 (Fig. 1) was the most selective compound in the synthesized series [17]. A series of thiazole acetamide derivatives were synthesized and potent inhibitory activities were observed in compound St.2 (Fig. 1) with an IC_50_ value of 9.01 µM [18]. Another series of 2-(trimethoxyphenyl) thiazoles derivatives were synthesized and compound St.3 (Fig. 1) was one of the most potent compounds against COX enzymes [19]. Many structures containing thiazole were synthesized and evaluated as COX inhibitors with significant activities [20, 21]. In our last works, we attempted to synthesis a series of phenyl-heterocycle-carboxamide and evaluate their COX inhibitory activities, phenyl were substituted with electron withdrawing atoms like F and Cl atoms on different positions on the phenyl ring [22, 23], However, among these series it was observed that almost all of these compounds were active against both COX1 and COX2, with low selectivity ratio, compound **St.4** (Fig. 1) was the most selective compound with selectivity ratio 1.44 [22] and the selectivity were improved with compound **St.5** (Fig. 1) when the carboxamide-phenyl bearing dimethoxy groups the ratio was 4.63 [23].

Depending on the previous data, the current work aimed to synthesize a novel series of *methoxyphenyl* thiazole carboxamide derivatives (**2a–2i**) and evaluate their activities on COX enzymes and their cytotoxicity on cancer cell lines (Huh7, MCF-7, and HCT116). Finally, molecular docking studies were performed to justify the possible binding interactions between the COX enzymes and our compounds. Thus, developing and discovering new selective no-toxic COX inhibitors is warranted. The efforts here in our study to investigate the binding patterns of native and newly discovered agents within the binding domain of COX-1, COX-2, bacterial, and fungal enzymes along with previous studies could finally pull off that supreme goal.

## Methods

### Chemistry

All chemicals were purchased from Alfa Aesar (Ward Hill, Massachusetts, USA) and Sigma-Aldrich (Schnelldorf, Germany). Melting points were determined with an SMP-II Digital Melting Point Apparatus and were uncorrected. ^1^H-NMR and ^13^C-NMR spectra were recorded in DMSO-d_6_ and were performed on Bruker 500 MHz-Avance III High-Performance Digital FT-NMR spectrometer at the Faculty of Science, Department of Chemistry, The University of Jordan, Jordan. All chemical shifts were recorded as d (ppm). High-resolution mass spectra data (HRMS) were collected using a Waters LCT Premier XE Mass Spectrometer, i.e., a high sensitivity orthogonal acceleration time-of-flight instrument. Using ESI (+) method, i.e., the instrument was coupled to an EQUITY Ultra Performance Liquid Chromatography system (Waters Corporation, Milford, MA, USA) at Pharmacy Faculty Gazi University Ankara-Turkey.

### General procedure for the synthesis of thiazole carboxamide (2a-2i)

2-(4-Methoxyphenyl)thiazole-4-carboxylic acid (2.34 mmol) was dissolved in dichloromethane (DCM; 20 mL), and then DMAP (0.78 mmol), EDC (3.12 mmol) were added and allowed to stir under argon gas (inert gas) at room temperature for 30 min. Aniline derivative was then added, and the mixture was allowed to stir for 48 h. TLC monitored the reaction till the end reaction. The excess aniline was washed by extraction with HCl. The reaction mixture was dried under reduced pressure using a rotary evaporator. The residues of the obtained product were purified by column chromatography using an appropriate solvent system [24–26].

### 2-(4-methoxyphenyl)-N-phenylthiazole-4-carboxamide (2a)

The product was purified by silica gel column chromatography using *n*-hexane: ethyl acetate solvent system (4:1). Solid product M.P. 138–140 °C, Yield: 74.5%. IR (FTIR/FTNIR-ATR): 1675.08 cm^−1^ amide carbonyl (C=O). HRMS (m/z): [M + H]^+^ calcd for C_17_H_14_N_2_O_2_S 311.0650, found 311.0658. ^1^H NMR (DMSO-d_6_) δ: 10.19 (1H, s, NH), 8.39 (1H, s, Ar–H), 8.10 (2H, d, *J* = 8.5 Hz, Ar–H), 7.87 (2H, d, *J* = 8 Hz, Ar–H), 7.39 (2H, t, *J* = 8 Hz, Ar–H), 7.15–7.10 (3H, m, Ar–H), 3.86 (3H, s, –OCH_3_). ^13^C NMR (DMSO-d_6_) δ ppm: 167.73, 161.80, 159.52, 150.68, 138.83, 129.11, 128.82, 125.65, 124.68, 124.41, 120.96, 115.01, 55.93.

### N-(3,4-dimethoxyphenyl)-2-(4-methoxyphenyl)thiazole-4-carboxamide (2b)

The product was purified by silica gel column chromatography using n-hexane: ethyl acetate solvent system (4:1). Solid product M.P. 123.5–1.24.5 °C, Yield: 92.4%. IR (FTIR/FTNIR-ATR): 1645.76 cm^−1^ amide carbonyl (C = O). HRMS (m/z): [M + H]^+^ calcd for C_19_H_18_N_2_O_4_S 371.1066, found 371.0882. ^1^H NMR (DMSO-d_6_) δ: 10.06 (1H, s, NH), 8.34 (1H, s, Ar–H), 8.10 (2H, d, *J* = 8.5 Hz, Ar–H), 7.54 (1H, s, Ar–H), 7.47 (1H, d, *J* = 8.5 Hz, Ar–H), 7.11 (2H, d, *J* = 8.5 Hz, Ar–H), 6.96 (1H, d, *J* = 9 Hz, Ar–H), 3.86 (3H, s, -OCH_3_), 3.79 (3H, s, -OCH_3_), 3.76 (3H, s, -OCH_3_). ^13^C NMR (DMSO-d_6_) δ ppm: 167.69, 161.79, 159.19, 150.83, 148.96, 145.82, 132.34, 128.81, 125.67, 124.28, 115.01, 112.84, 112.31, 106.06, 56.17, 55.93.

### N-(3,5-dimethoxyphenyl)-2-(4-methoxyphenyl)thiazole-4-carboxamide (2c)

The product was purified by silica gel column chromatography using n-hexane: ethyl acetate solvent system (3.5:1.5). Solid product M.P. 156–158 °C, Yield: 66.7%. IR (FTIR/FTNIR-ATR): 1664.89 cm^−1^ amide carbonyl (C = O). HRMS (m/z): [M + H]^+^ calcd for C_19_H_18_N_2_O_4_S 371.1066, found 371.1044. ^1^H NMR (DMSO-d_6_) δ: 10.10 (1H, s, NH), 8.38 (1H, s, Ar–H), 8.10 (2H, d, *J* = 8.5 Hz, Ar–H), 7.19 (2H, s, Ar–H), 7.11 (2H, d, *J* = 8.5 Hz, Ar–H), 6.30 (1H, s, Ar–H), 3.86 (3H, s, -OCH_3_), 3.76 (6H, s, -OCH_3_).^13^C NMR (DMSO-d_6_) δ ppm: 167.77, 161.82, 160.89, 159.52, 150.57, 140.51, 128.85, 125.62, 124.81, 115.00, 99.08, 96.50, 55.93, 55.65.

### N-(2,5-dimethoxyphenyl)-2-(4-methoxyphenyl)thiazole-4-carboxamide (2d)

The product was purified by silica gel column chromatography using n-hexane: ethyl acetate solvent system (4:1). Solid product M.P. 139.5–141.5 °C, Yield: 70.1%. IR (FTIR/FTNIR-ATR): 1682.68 cm^−1^ amide carbonyl (C=O). HRMS (m/z): [M + H]^+^ calcd for C_19_H_18_N_2_O_4_S 371.1066, found 371.0918. ^1^H NMR (DMSO-d_6_) δ: 9.87 (1H, s, NH), 8.42 (1H, s, Ar–H), 8.04 (1H, s, Ar–H), 7.97 (2H, d, *J* = 7 Hz, Ar–H), 7.12 (2H, d, *J* = 8.5 Hz, Ar–H), 7.05 (1H, d, *J* = 9 Hz, Ar–H), 6.69 (1H, d, *J* = 8.5 Hz, Ar–H), 3.92, 3.85, 3.74 (9H, s, -OCH_3_). ^13^C NMR (DMSO-d_6_) δ ppm: 168.14, 161.92, 158.53, 153.63, 150.14, 143.00, 128.51, 128.03, 125.25, 124.89, 115.26, 112.11, 108.38, 106.57, 57.06, 55.93, 55.83.

### N-(2,4-dimethoxyphenyl)-2-(4-methoxyphenyl)thiazole-4-carboxamide (2e)

The product was purified by silica gel column chromatography using n-hexane: ethyl acetate solvent system (3:2). Solid product M.P. 132.5–134 °C, Yield: 78.0%. IR (FTIR/FTNIR-ATR): 1674.82 cm^−1^ amide carbonyl (C=O). HRMS (m/z): [M + H]^+^ calcd for C_19_H_18_N_2_O_4_S 371.1066, found 371.0731. ^1^H NMR (DMSO-d_6_) δ: 9.67 (1H, s, NH), 8.36 (1H, s, Ar–H), 8.14 (1H, d, *J* = 8.5 Hz, Ar–H), 7.98 (2H, d, *J* = 8 Hz, Ar–H), 7.12 (2H, d, *J* = 8 Hz, Ar–H), 6.72 (1H, s, Ar–H), 6.58 (1H, d, *J* = 8.5 Hz, Ar–H), 3.94, 3.85, 3.78 (9H, s, -OCH_3_).^13^C NMR (DMSO-d_6_) δ ppm: 167.97, 161.86, 158.32, 157.12, 150.60, 150.48, 128.52, 125.37, 124.25, 121.34, 120.62, 115.21, 104.76, 99.34, 56.65, 55.92, 55.81.

### 2-(4-methoxyphenyl)-N-(3,4,5- trimethoxyphenyl)thiazole-4-carboxamide (2f)

The product was purified by silica gel column chromatography using n-hexane: ethyl acetate solvent system (3.5:1.5). Solid product M.P. 174–176 °C, Yield: 66.5%. IR (FTIR/FTNIR-ATR): 1671.44 cm^−1^ amide carbonyl (C=O). HRMS (m/z): [M + H]^+^ calcd for C_20_H_20_N_2_O_5_S 401.0955, found 401.0946. ^1^H NMR (DMSO-d_6_) δ: 10.08 (1H, s, NH), 8.36 (1H, s, Ar–H), 8.10 (2H, d, *J* = 8.5 Hz, Ar–H), 7.35 (2H, s, Ar–H), 7.11 (2H, d, *J* = 8 Hz, Ar–H), 3.86 (3H, s, –OCH_3_), 3.80 (6H, s, –OCH_3_), 3.66 (3H, s, –OCH_3_).^13^C NMR (DMSO-d_6_) δ ppm: 167.79, 161.82, 159.35, 153.12, 150.65, 134.96, 134.35, 128.83, 125.62, 124.61, 115.01, 98.62, 60.57, 56.27, 55.93.

### N-(4-chloro-2,5-dimethoxyphenyl)-2-(4-methoxyphenyl)thiazole-4-carboxamide (2g)

The product was purified by silica gel column chromatography using n-hexane: ethyl acetate solvent system (3:2). Solid product M.P. 172.5–174.5 °C, Yield: 57.1%. IR (FTIR/FTNIR-ATR): 1666.92 cm^−1^ amide carbonyl (C = O). HRMS (m/z): [M + H]^+^ calcd for C_19_H_17_ClN_2_O_4_S 405.0676, found 405.0455. ^1^H NMR (DMSO-d_6_) δ: 9.84 (1H, s, NH), 8.41 (1H, s, Ar–H), 8.26 (1H, s, Ar–H), 7.96 (2H, d, *J* = 8.5 Hz, Ar–H), 7.24 (1H, s, Ar–H), 7.12 (2H, d, *J* = 8.5 Hz, Ar–H), 3.94 (3H, s, -OCH_3_), 3.85, 3.83 (6H, s, -OCH_3_).^13^C NMR (DMSO-d_6_) δ ppm: 168.22, 161.93, 158.59, 149.90, 148.80, 143.11, 128.52, 126.92, 125.21, 125.07, 115.62, 115.24, 113.50, 105.13, 57.44, 56.81, 55.93.

### N-(4-(tert-butyl)phenyl)-2-(4-methoxyphenyl)thiazole-4-carboxamide (2h)

The product was purified by silica gel column chromatography using n-hexane: ethyl acetate solvent system (3:2). Solid product M.P. 135–137 °C, Yield: 75.6%. IR (FTIR/FTNIR-ATR): 1681.58 cm^−1^ amide carbonyl (C=O). HRMS (m/z): [M + H]^+^ calcd for C_21_H_22_N_2_O_2_S 367.1480, found 367.1301. ^1^H NMR (DMSO-d_6_) δ: 10.12 (1H, s, NH), 8.37 (1H, s, Ar–H), 8.09 (2H, d, *J* = 8.5 Hz, Ar–H), 7.77 (2H, d, *J* = 8.5 Hz, Ar–H), 7.40 (2H, d, *J* = 8.5 Hz, Ar–H), 7.11 (2H, d, *J* = 8.5 Hz, Ar–H), 3.86 (3H, s, –OCH_3_), 1.30 (9H, s, t-butyl).^13^C NMR (DMSO-d_6_) δ ppm: 167.70, 161.79, 159.38, 150.79, 146.76, 134.24, 128.82, 125.72, 125.67, 124.51, 120.72, 115.01, 55.93, 34.56, 31.67.

### 2-(4-methoxyphenyl)-N-(4-(thiophen-2-yl)phenyl)thiazole-4-carboxamide (2i)

The product was purified by silica gel column chromatography using n-hexane: ethyl acetate solvent system (4:1). Solid product M.P. 135–137 °C, Yield: 49.8%. IR (FTIR/FTNIR-ATR): 1675.17 cm^−1^ amide carbonyl (C=O). HRMS (m/z): [M + H]^+^ calcd for C_21_H_16_N_2_O_2_S_2_ 393.0577, found 393.0569. ^1^H NMR (DMSO-d_6_) δ: 10.31 (1H, s, NH), 8.41 (1H, s, Ar–H), 8.11 (2H, d, *J* = 8.5 Hz, Ar–H), 7.94 (2H, d, *J* = 8.5 Hz, Ar–H), 7.69 (2H, d, *J* = 8.5 Hz, Ar–H), 7.51 (2H, d, *J* = 9 Hz, Ar–H), 7.14–7.11 (3H, m, Ar–H), 3.86 (3H, s, –OCH_3_).^13^C NMR (DMSO-d_6_) δ ppm: 167.85, 159.53, 150.58, 143.63, 138.32, 128.93, 128.85, 126.19, 126.16, 125.60, 124.86, 123.61, 121.36, 121.34, 115.07, 115.03, 55.95.

## Biological methods

### Biological COX assay method

The COX-1 and COX-2 enzyme inhibitory activities were investigated using the COX (human) Inhibitor Screening Assay Kit (supplied by Cayman Chemicals, Ann Arbor, MI, USA). The preparation of all reagents and evaluation procedures were conducted according to the manufacturer's instructions. In brief, two concentrations of the compounds and ketoprofen (concentration range: 40 and 5 μM) dissolved in a minimum quantity of dimethylsulfoxide (DMSO) were incubated with a mixture of both enzymes, COX-1 or COX-2. The reaction was started by adding 50 μL of AA followed by incubation at 37 °C for precisely 30 s. Then, the reaction was sealed by adding 30 μL of stannous chloride solution to each reaction test tube and followed by incubation for 5 min at room temperature. The produced PGF2a in the samples by COX reactions was quantified by enzyme-linked immunosorbent assay (ELISA). The 96-well plate was covered with plastic film and incubated for 18 h at room temperature on an orbital shaker. In the final steps, the plate was rinsed five times with the washed buffer followed by the addition of Ellman’s reagent (200 μL) and incubated for about 60–90 min at room temperature until the absorbance of the B^o^ well is in the range of 0.3–0.8 at 405 nm. The plate was then read by an ELISA plate reader Unilab microplate reader 6000 (Geneve, Switzerland). The inhibitory percentage was measured for the tested concentrations in comparison with the control [17, 27].

### Cell culture

Cell lines from different cancer types (Huh7; liver, MCF7; breast and HCT116; colon) were grown in Dulbecco's Modified Eagles Medium (DMEM) supplemented with 10% fetal bovine serum (FBS) (Invitrogen, GIBCO) and 1% non-essential amino acids (Invitrogen, GIBCO) and 100 units/mL of penicillin and streptomycin. Cells were maintained in a humidified incubator with 5% CO_2_, at 37 °C [28].

### Sulforhodamine B (SRB) assay

Cells were plated in 96-well plates (2000–3000 cells/well) in 150 µL/well complete DMEM. 24 h later, cells were treated with the compounds at increasing concentrations (40–2.5 µM) in triplicate. After 72 h, cells were washed with PBS and fixed with 10% (v/v) trichloroacetic acid (MERCK). SRB staining was performed as described previously and absorbance measurements were obtained at 515 nm using a plate reader [29]. IC_50_ values were calculated with the use of dose–response curves (percent growth inhibition vs drug concentration) generated for each compound.

### Molecular docking studies

Molecular docking studies were conducted to investigate the best binding pose of the docked ligands inside the binding pocket of the receptor so that could interpret the reported biological results by setting the ligand-receptor interaction patterns. Docking studies were preceded by following a general procedure starting from ligand drawing and preparation, receptor preparation, and grid generation, which was followed by performing XP-Glide docking studies.

### Ligand drawing and preparation

At first, the ligands selected for docking studies were drawn using the Graphical User Interface of Maestro- Schrödinger 12.1 [30]. Using the LigPrep, the ligands were then prepared at the OPLS2005 force field and target pH (7.0 ± 2.0) protonation state [17].

### Protein preparation and grid generation

Three-dimension crystal structures of both human cyclooxygenase proteins COX1 (PDB ID 3KK6) and COX2 (PDB ID 5KIR) were applied for molecular docking studies downloaded from the Brookhaven protein data bank (PDB; http://www.rcsb.org/pdb). Those two PDB ID codes (3KK6 and 5KIR) were exercised and optimized in our previous work [17] that had a high resolution (e.g., resolutions were 2.75 Å and 2.70 Å, respectively) and showed relevant binding orientations. After that, the downloaded crystal structures were prepared and refined using the Protein Preparation Wizard of Schrödinger-Maestro 12.1 which involved adding hydrogens to heavy atoms, filling missing side chains using Prime, assigning binding orders and charges, deleting all water molecules beyond 5 Å from the het group. Then, protein structures were minimized using the OPLS_2005 force field [31]. Finally, the receptor grids were generated following the default setup.

### Glide extra-precision (XP) ligand docking

Using the Glide of Schrödinger-Maestro 12.1, XP flexible ligand docking was performed within partial charge cutoff and the Van der Waals scaling factor was selected to be 0.15 and 0.80, respectively, for ligand atoms [32]. On energy minimized pose, the final scoring was carried out, and as Glide score was displayed. The docked pose showed the lowest Glide score was considered the best pose and was recorded for each docked ligand. Those best-docked poses were exported to the PLIP server to investigate the binding mode precisely. The PyMOL program 2.5.2 was applied to visualize the final results [33, 34].

### Free energy calculations using Prime MM-GBSA

In order to estimate the energy binding affinities of the likely ligand binding modes within the docking results, the Prime MM-GBSA approach (Molecular mechanics‐Generalized Born Surface Area) of Schrodinger was employed following the default parameter settings [35]. In this module, a local optimization through molecular mechanics (MM) in Prime was applied, followed by minimizing the energies of docked ligand-receptor complexes with a generalized Born surface area (GBSA) continuum solvent model under a force field of OPLS-AA (2005). The binding free energy is calculated as shown in the equation supported in the Additional file 1 [36].

### Density functional theory analysis

As the biological activity of drug-like molecules is mainly driven by the electronic pattern, the geometry of the best binding pose regarding docked ligands was exported to Maestro- Schrödinger 12.1 version and optimized in the Jaguar panel using Lee–Yang–Parr correlation functional (B3LYP) theory with a 6-31G** basis set [37, 38] and Becke’s three-parameter exchange potential [39]. The atomic electrostatic potential charges (EPS) and surfaces (molecular orbital, density, potential) were applied to compute the HOMO and LUMO, subsequently. The region of small molecules which could donate electrons during the complex formation is proposed by HOMO energy, while the capacity of small molecules to accept electrons from the partner protein is signified by LUMO energy. The HOMO–LUMO gap energy points to the electronic excitation energy, which is the different energies between HOMO and LUMO [40]. HOMO–LUMO gap energy is a substantial parameter to estimate molecular stability and reactivity [41].

### Ligand-based ADME/Toxicity prediction

The QikProp module of Maestro-Schrodinger version 12.1 (2021-3 release) was utilized to predict precisely both pharmacokinetically relevant properties and physicochemical significant descriptors. It’s easy to use, accurate, and quick prediction software designed to calculate particular descriptors related to absorption, distribution, metabolism, excretion, and toxicity (ADME-T) [42].

### Statistical analyses

Determination of the biological activities was carried out in triplicate for each sample. The obtained results were presented as means ± standard deviation (SD).

## Results and discussion

### Chemistry

**Scheme 1:**
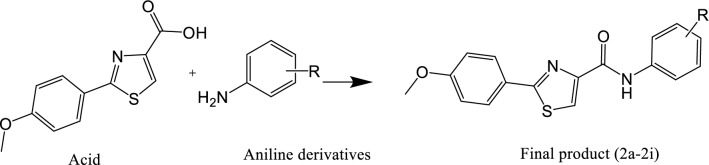
Acid + appropriate aniline derivatives, stirred in 20 mL DCM, then DMAP and EDC were added under inert argon gas and stirred for 48 h

The methoxyphenyl thiazole carboxamide derivatives (**2a–2i)** were synthesized as outlined in Scheme 1. Here, following our previously published data, the methoxy phenyl substituent is used as a fixed and bulky group aiming to utilize its favorable interactions, geometry, and fitting capacity shown within the binding pockets of COX enzymes [22, 23]. Moreover, to enhance the electron density within the linker region, the heterocyclic thiazole ring was incorporated so proposed to enhance the interaction pattern in terms of hydrophobic and π–π interactions. Furthermore, within the phenyl amide ring, large bulky groups also are used at various positions to examine the optimal capacity and the topological structure of the binding pocket.

Regarding the synthesis strategy, the coupling reaction was afforded to form the final products (**2a–2i**) by EDCI and DMAP as activating agents and covalent nucleophilic catalysts, respectively. After the coupling step, they reacted with the aniline derivatives. The products were purified using solvent systems (n-hexane: ethyl acetate). All compounds showed sharp bands for carbonyl amide around 1670 cm^−1^. The HRMS values of all synthesized compounds confirmed their molecular weight. The ^1^H-NMR peaks confirmed the synthesis of these products, a single peak of one proton for N–H amide in the range of 9.67–10.31 ppm was observed for each compound. Multiple signals in the aromatic area were observed, and single peaks integrated for the protons of methoxy groups were observed around 3.86–3.76 ppm, as well as a signal peak of 9 protons, was observed for the compound with a tret-butyl substituent at 1.30. The ^13^C-NMR spectrum showed a C signal of carbonyl around 167 ppm.

### In vitro COX-1 and COX-2 inhibition assay

**Fig. 2:**
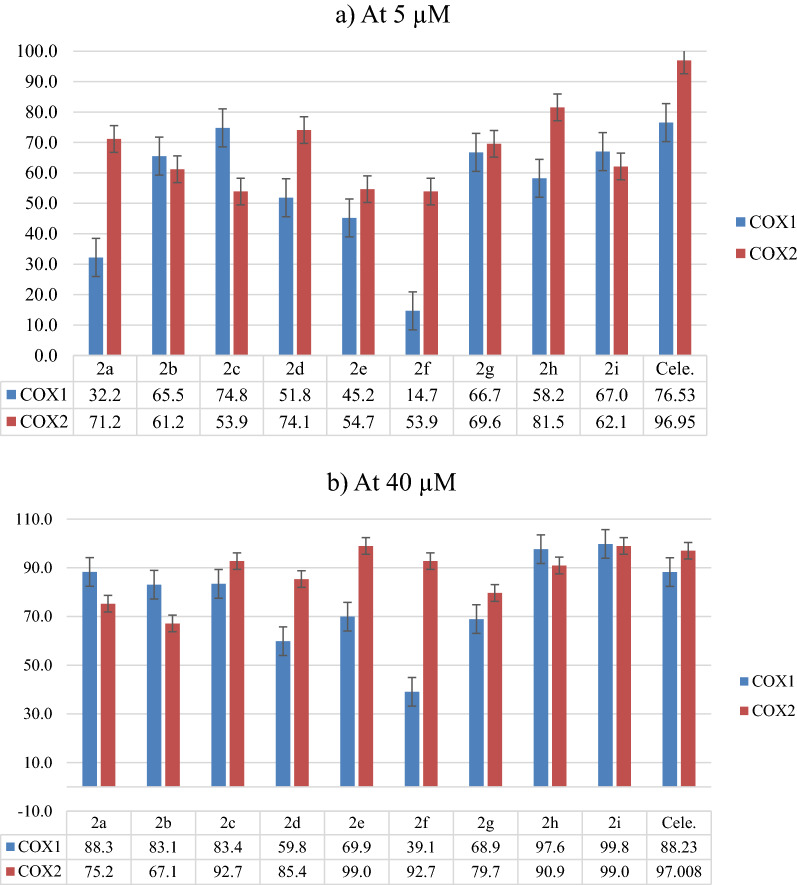
The inhibitory percentage of the synthesized compound in comparison with the positive control celecoxib at two concentrations **a** 5 µM and **b** 40 µM

**Fig. 3:**
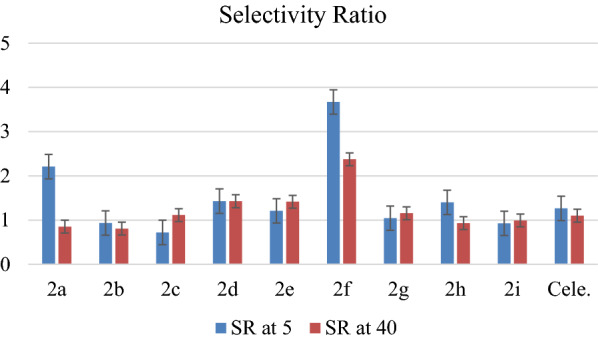
The Selectivity ratio (SR) of the synthesized compound in comparison with the positive control celecoxib at two concentrations 5 µM and 40 µM

All compounds were evaluated for inhibition assay on COX-1 and COX-2 enzymes using the COX-1 (human) Inhibitor Screening Assay Kit and COX-2 (human) Inhibitor Screening Assay Kit (Cayman Chemical Company, Ann Arbor, MI, USA). At the used concentrations, all synthesized compounds showed potent inhibitory activities at both COX enzymes, as shown in Fig. 2. At 5 µM concentrations, all compounds showed an inhibitory percentage higher than 53.9% against the COX-2 enzyme, while the inhibitory percentage against the COX-1 enzyme for all compounds was in the range of 32.2–74.8%. However, the most potent compound against the COX-2 enzyme was 2 h, with an inhibitory percentage of 81.5% in comparison with the positive control celecoxib value of 96.9%. All of the tested compounds showed selectivity toward the COX-2 enzyme except **2b, 2c,** and **2i**. The most selective compound was **2f,** in which at 5 and 40 µM concentrations the inhibitory percentages against COX-2 were 53.9 and 92.7%, respectively. While COX-1 inhibitory percentages were 14.7 and 39.1%, respectively, Actually, the selectivity of this compound is due to the presence of the bulky functional group trimethoxyphenyl. Moreover, the selectivity ratio (SR) were calculated and present in Fig. 3, it was clear that compound **2f** was the most selective to both concentrations (5 and 40 µM), with values of 3.67 and 2.37 respectively.

### Cytotoxic evaluation

Methoxyphenyl thiazole carboxamide derivatives (**2a–2i)** were screened against hepatocellular (Huh7), breast (MCF7), and colon carcinoma (HCT116) cells using the SRB assay [43]. The IC_50_ values, defined as the half-maximal inhibitory concentration for cell growth, are summarized in Table 1. The results revealed that all of the synthesized Methoxyphenyl thiazolecarboxamide derivatives did not exhibit antiproliferative activity with IC_50_ values higher than 40 µM concentration except compounds **2f and 2e,** and this means that these compounds have no cytotoxic activities against the used cell lines at the effective dose. There is strong relationship between the COX enzyme and tumer overexprasion, the COX-2 enzyme is usually overexpressed in severl sorts of human cancers, and the biological studies consistently explained that COX-2 inhibitors compounds can inhibit the tumor progression and metastasis in several animal models of cancer [44, 45]. However, compounds like **2f** with COX inhibitory and anticancer activities could be a promising agents for both targets.

**Table 1:** The IC_50_ of the synthesized compound against three different cancer cell lines

		IC_50_		
Compound Code	R	Huh7	MCF-7	HCT116
**2a**	H	> 40	> 40	> 40
**2b**	3,4-OCH_3_	> 40	> 40	> 40
**2c**	3,5-OCH_3_	NI	NI	NI
**2d**	2,5-OCH_3_	NI	NI	NI
**2e**	2,4-OCH_3_	NI	> 40	35.70 ± 0.79
**2f**	3,4,5-OCH_3_	**17.47 ± 0.85**	NI	**14.57 ± 0.93**
**2g**	2,5-OCH_3_, 4-CL	NI	NI	NI
**2h**	4-t-butyl	> 40	NI	> 40
**2i**	4-thiophene	NI	NI	NI

NI: no inhibition, > 40: inhibition was observed at concentrations higher than 40 µM

### Molecular docking studies

The geometry and sequence identity of both cyclooxygenase isoforms (COX-1 and COX-2) are analogous, and the selectivity of the newly discovered non-steroidal anti-inflammatory drugs (NSAIDs) could be boosted by taking advantage of the presence of VAL523 amino acid within the binding site of COX-2 isozyme instead of ILE523 amino acids in COX-1 isozyme. This minimal change led to critical modifications in the geometry and electron behavior of the COX-2 isoform through unlocking a secondary binding pocket and the polar amino acid ARG-513 which is become uncovered. Filling optimally this newly opened secondary binding pocket and interacting with ARG513 is supposed to ameliorate COX-2 selectivity [46]. Investigating the interaction profile of selected ligands within the binding pockets of both crystallized human COX-1 and COX-2 isozymes is supposed to interpret the recorded biological activity and selectivity results alongside enriching and confirming the previous studies.

**Fig. 4:**
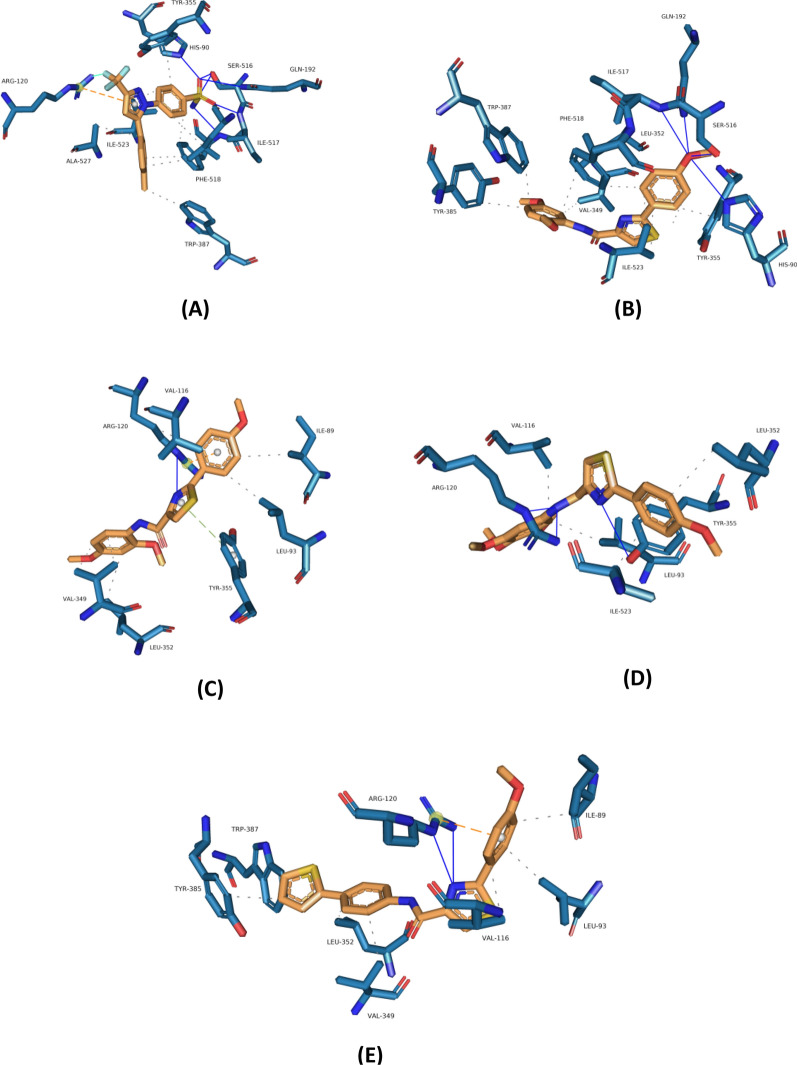
Crystal binding mode of (**A**) celecoxib, and predicted binding orientations of (**B**) 2d, (**C**) 2e, (**D**) 2f, and **E** (2i) visualized in COX-1 active site (PDB code 3KK6); ligands are shown in orange and amino acids are shown in blue. Only the interacting residues and their interaction patterns are shown

**Fig. 5:**
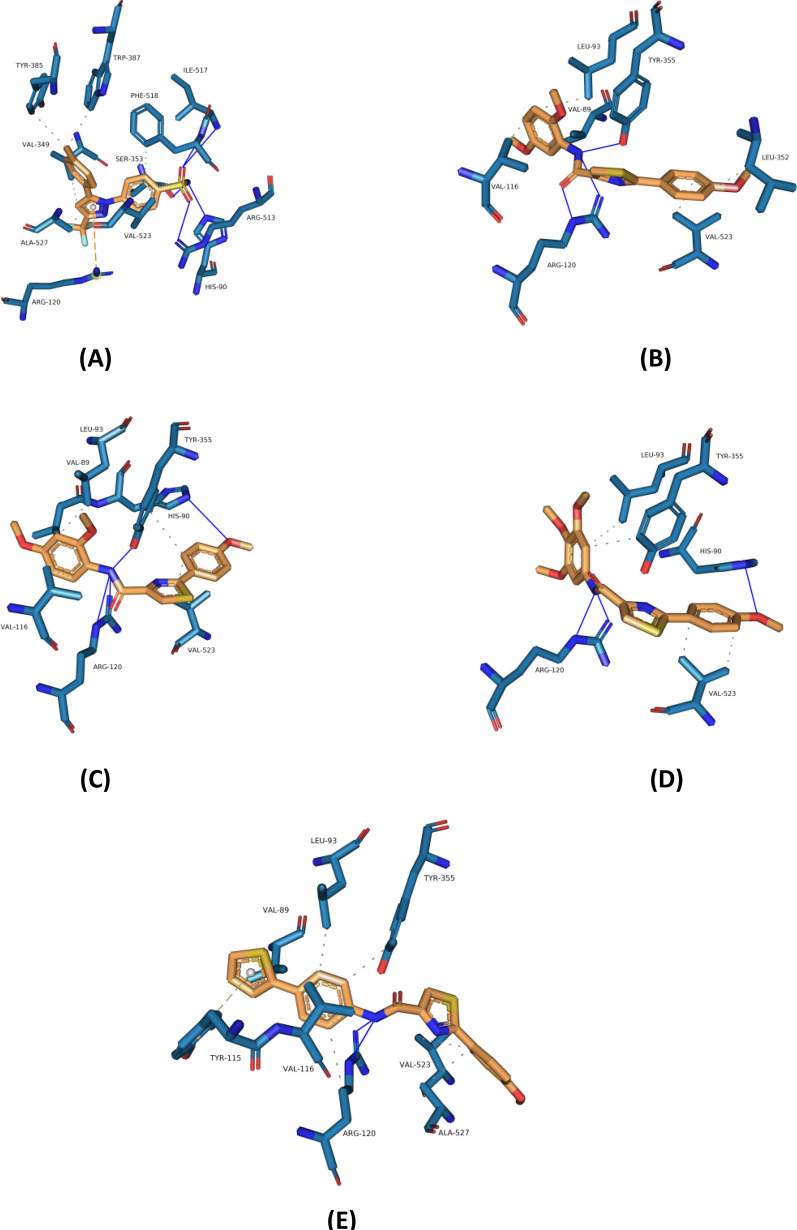
Crystal binding mode of (**A**) celecoxib, and predicted binding orientations of (**B**) 2d, (**C**) 2e, (**D**) 2f, and **E** (2i) visualized in COX-2 active site (PDB code 5KIR); ligands are shown in orange and amino acids are shown in blue. Only the interacting residues and their interaction patterns are shown

Out of the nine biologically tested ligands, four ligands (**2d**, **2e**, **2f**, and **2i**) were selected for docking studies. These ligands showed the best inhibition profile at both low (5 μM) and high (40 μM) concentrations besides keeping their selectivity toward COX-2 isozyme at both tested concentrations. These advanced results are boosted by revealing negligible cytotoxicity except **2f** which showed moderate toxicity. The obtained results of docking analysis regarding celecoxib, selected as a positive control, and the four selected ligands docked to COX-1 and COX-2 human crystallized receptors are summarized in Figs. 4 and 5, respectively, (higher resolution of figures were provided in the Additional file 1: Figs S10-S19). The XP-Glide docking scores, which is an empirical scoring function, recorded for the docking analysis are summarized in Table 2. The docking score is calculated by considering many parameters supposed to modulate ligand-receptor binding affinity including force field (electrostatic, van der Waals) contributions and terms penalizing or rewarding interactions [47].

**Table 2:** Docking score of the designed ligands with the COX 1 and COX 2 receptors and the free energy calculation (ΔG_bind_) of ligand-drug complexes using Prime/MM-GBSA

Ligands	COX-1		COX-2	
	Docking score	ΔG_bind_	Docking score	ΔG_bind_
2d	− 5.527	− 49.60	− 6.582	− 57.06
2e	− 4.996	− 63.45	− 5.674	− 65.95
2f	− 5.308	− 57.90	− 5.492	− 58.99
2i	− 5.239	− 64.56	− 6.004	− 67.70
Celecoxib	− 10.86	− 73.89	− 11.28	− 80.18

Within the binding pocket of the COX-1 isozyme, celecoxib showed a congruent interaction pattern to previously published studies. As shown in Fig. 4A, celecoxib locates at ideal hydrogen bond interaction ranges to the amino acid residues HIS-90, GLN-192, SER-516, ILE-517, and PHE-518. Also, the diazole ring and the tri-fluor group interact optimally with the guanidine functional group of ARG-120 forming a pi-cation interaction and a halogen bond. Those advanced interactions are assisted by further hydrophobic interactions within the binding site. Compared to celecoxib, examining the supposed interaction profile of **2d**-compound showed a comparable interaction profile, except for losing the two advanced interactions with ARG-120 residue inside the binding site. **2e**-compound interacted ideally with the ARG-120 amino acid residue forming hydrogen, hydrophobic, and pi-cation interactions. The compound also formed bi-stacking with TYR-355 residue beside further hydrophobic interactions. The lowest recorded inhibition biological activity of **2f**-compound toward COX-1 enzyme could be explained through forming lesser positive binding contribution interactions within the binding pocket. The non-ability to localize the aromatic rings optimally inside the binding site led to the forfeit of any pi-stacking or pi-cation interactions as a result of existing steric clashes that deprived the acquired advanced interactions. The inhibition activity of Compound-**2i** could be explained by creating two hydrogen bonds and pi-cation interaction with ARG-120 amino acid residue, besides other hydrophobic interactions, within the COX-1 binding site. These recorded results and the biological activity were reflected by consistent docking scores. Displaying a superior docking score and inhibition activity, especially at the lower applied concentration, of celecoxib over our newly designed ligands is considered an advantage for the tested ligands regarding the selectivity toward COX-2 isozyme.

Concerning the molecular docking analysis within the binding pocket of COX-2, celecoxib (Fig. 5A) displayed the ability to form various advantageous interactions resulting in opening a secondary binding pocket and unveiling a new binding residue (ARG-513), this newly added hydrogen bond is considered a preferable interaction toward COX-2 selectivity over COX-1. Other favorable interactions include hydrogen bonds with HIS-90, SER-353, ILE-517, and PHE-518 and a pi-cation interaction with ARG-120 residue. Celecoxib is stabilized more via forming a lot of hydrophobic interactions with the surrounding residues inside the binding site. This XP-glide docking analysis of celecoxib is inconsistent with the previously recorded data in the literature. Compound-**2d, 2e,** and **2f** showed comparable binding geometry and interaction profiles which explain their analogous biological activity. However, the absence of that critical binding interaction with ARG-513, they can form other positive contributing hydrogen bonds with two or three amino acids residues like ARG-120, HIS-90, and TYR-355. These polar interactions are boosted by other favorable hydrophobic interactions that assisted in fitting the binding pocket more. As observed here, compared to 2e and 2d candidates, the existence of three large functional groups like –O-CH_3_ in the 2f compound led to boosting the selectivity forward the COX2 isozyme over COX1. Hence, due to replacing the ILE-523 in COX1 with the VAL-523 in COX-2, the large binding pocket of the COX2 enzyme is better occupied and fitted by the tri-methoxy phenyl portion than that observed in COX1. The tri-methoxy group couldn’t fit optimally within the COX1 isozyme and displayed some steric clashes with the surrounding residues, reflected by dropping in the COX1 inhibition activity. Replacing the substituted phenyl ring with a thiophene ring in compound-**2i** led to creating some differences regarding the geometry and interaction profile within the binding site of COX-2. Compared to other docked ligands, the methoxyphenyl ring is flipped down toward VAL-523 amino acid residue which resulted in forming additional hydrophobic interactions with VAL-523 and ALA-527 residues. The absence of substituents at the thiophene ring compared to the substituted phenyl ring led to being in a favorable geometry and binding range with TYR-115 to form a new pi-stacking interaction.

However, the molecular docking score parameter is considered an effective indicator tool within drug-discovery applications, it’s not a totally precise tool and highly recommended to examine it along with the structural criteria and interaction profiles obtained for the docked analogs, especially in the case of obtaining nearby docking scores. The docking scoring process mainly utilizes simple scoring fuctions which aim to gather the leading interactions but may ignore others [48, 49]. Thus, as observed in Table 2, the calculated docking scores were not totally parallel but related to that observed in the biological assay. So, in terms of potency and selectivity, the docking scores did not differentiate between the candidates properly but were just presented in a close range, similar to that observed in the biological assay. The docking scores for the thiazole carboxamide derivatives ranged from − 5.49 to − 6.58 for COX2 and ranged from − 04.99 to − 5.52 for COX1 whilst the docking scores recorded for the celecoxib were − 11.28 and − 10.86 for the COX2 and COX1, respectively. The comparable, sometimes superior, COX-2 inhibition activity of our newly tested ligands over celecoxib is a vantage for these tested molecules. This advantage is highly supported by displaying lower docking scores and inhibition activity compared to celecoxib concerning COX-1 isozyme.

These promising results boost the selectivity behavior of the newly designed ligands toward COX-2 isozyme when compared to the well-known positive control (celecoxib).

### Analysis of Prime-MM/GBSA calculations

The stability of ligand-receptor complexes is mainly evaluated by calculating the Prime-MM/GBSA, which is considered the most precise parameter. Calculating the MM/GPSA involves considering various parameters that could manipulate the overall stability of ligand-receptor complexes. One of these critical parameters is the effect of solvent. Usually, the recorded Prime-MM/GBSA scores are highly correlated with the records defined experimentally however, the calculations of free energy are computationally demanding. As summarized in Table 2, the Prime-MM/GBSA values of COX-1 and COX-2 proteins in complex with the positive control (celecoxib) and the four selected ligands for molecular docking studies have been calculated. As evident, all the ligands/COX-2 complexes including celecoxib/COX-2 displayed lower ΔG_bind_ energies compared to ligands/COX-1 complexes which explained the higher binding affinity toward COX-2 isozyme. Celecoxib, 2d, 2e, 2f, and 2i showed ΔG_bind_ energy equal to − 73.89, − 49.60, − 63.45, − 57.90, and − 64.56 kcal mol^−1^ when complexed with COX-1 which decreased to − 80.18, − 57.06, − 65.95, − 58.99, and − 67.70 kcal mol^−1^_,_ respectively, when complexed with COX-2. Celecoxib showed the lowest ΔG_bind_ energy, which means a higher binding affinity, toward both COX-1 and COX-2 complexes compared to the newly designed ligands. These results are in parallel with the recorded XP-glide docking scores and the observed biological activity as explained previously.

### Density functional theory analysis

**Fig. 6:**
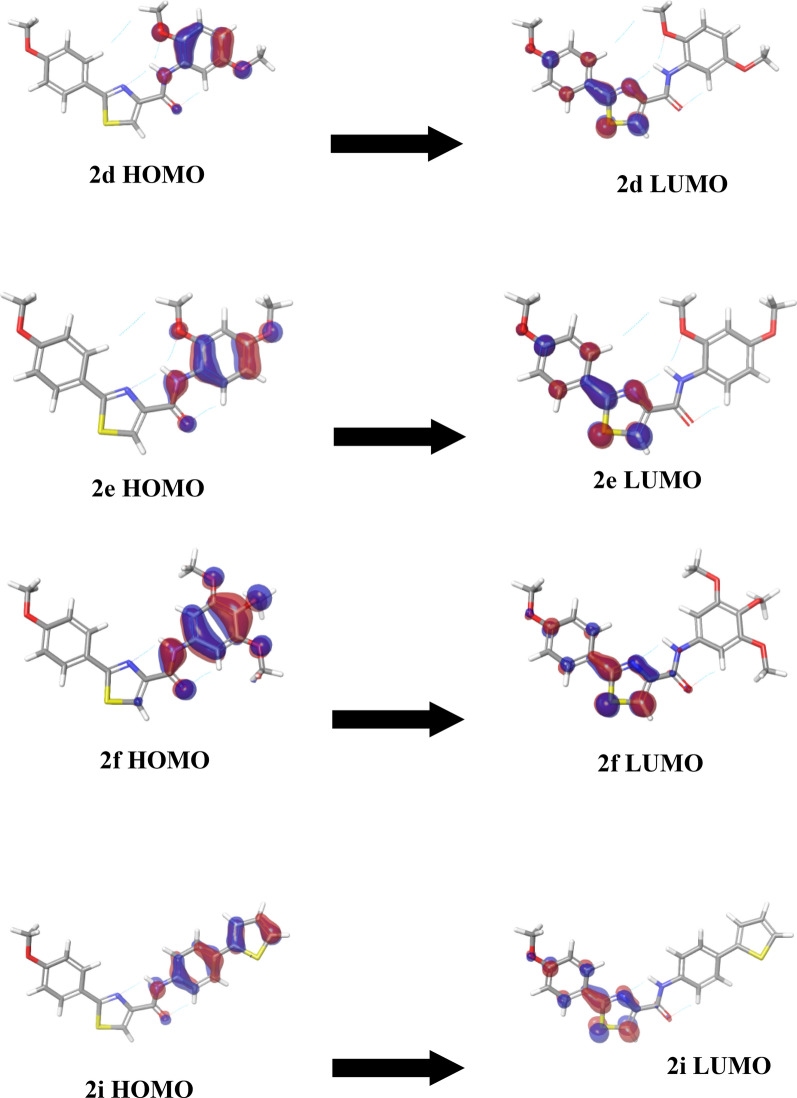
Plots of highest occupied molecular orbital (HOMO) and lowest unoccupied molecular orbital (LUMO) of compounds **2d, 2e, 2f,** and **2i**. The positive electron density has been shown in red color while the negative has been shown in blue

Based on the well-known role of HOMO–LUMO in stabilizing the interactions between receptor protein and ligand [40], using DFT, the chemical reactivity of our newly designed ligands was assessed by calculating the frontier orbital energy of both HOMO and LUMO besides calculating the HOMO–LUMO gap (Table 3). Furthermore, the distribution map of HOMO and LUMO surfaces were visualized for the tested candidates, as shown in Fig. 6 and Additional file 1: Fig. S20, to examine their constituent shape, positional, and symmetrical features. The negative electron density is presented in blue color while the red color indicates the positive electron density. The HOMO orbitals are the electron-rich orbitals so have a high capability to donate electrons while the LUMO orbitals have a high capability to accept electrons resuming from their poor electron feature. So, the HUMO and LUMO orbitals are almost associated with the nucleophilic and electrophilic attack, respectively. Thus, visualizing these orbital features is a valuable tool to explore the binding profile exceedingly besides resolving the atomic contribution of these orbitals within the target’s pocket. Visualizing the HOMO–LUMO surface maps demonstrated that the HOMO orbitals (regions of high electron density) existed over the phenyl amide fractions while the LUMO orbitals (regions of low electron density) presented within the phenyl thiazole fractions. These obtained data indicate that the phenyl amide regions could participate favorably with such interactions like hydrophobic, charge transfer, π–π, and π-stacking interactions, while the phenyl thiazole regions could associate properly with π-cationic interactions.

**Table 3:** Frontier orbital energies of the best seven lead compounds

Compound	HOMO (eV)	LUMO (eV)	HLG (eV)*
2a	− 0.21	− 0.06	− 0.15
2b	− 0.18	− 0.05	− 0.13
2c	− 0.20	− 0.06	− 0.14
2d	− 0.19	− 0.05	− 0.14
2e	− 0.18	− 0.05	− 0.13
2f	− 0.19	− 0.06	− 0.13
2g	− 0.19	− 0.06	− 0.13
2h	− 0.20	− 0.05	− 0.15
2i	− 0.19	− 0.06	− 0.13

*HOMO* highest occupied molecular orbital, *LUMO* lowest unoccupied molecular orbital, *HLG* HOMO–LUMO gap energy (the difference in orbital energy between HOMO and LUMO)

As shown in Table 3, summarizing the HOMO and LUMO energy maps, all our newly analyzed ligands displayed HOMO–LUMO energy gaps within − 13 and − 15 eV energy differences. By increasing the energy gap difference value, the chemical hardness increases while polarizability decreases as a result of demanding higher energy for the electron excitation process from the HOMO to the LUMO orbital. The lowest and the highest observed HOMO energy values were in compound-**2a** (− 0.21 eV) and compound-**2b** and **2e** (− 0.18 eV), respectively. The observed LUMO energy values were located in a close range (− 0.05 to − 0.06 eV). There is no clear relationship between the HOMO-energy, LUMO-energy, or HOMO–LUMO energy gap and biological activity. This could be explained by considering the site at which they exist and the direction at which they direct and therefore, what is the extent of their ability to interact well with the surrounding active orbitals within the protein binding pocket [50].

### ADME-T analysis

Aiming to investigate the drug-like activity of our newly designed molecules; selected ADME-T parameters were calculated utilizing the QikProp module of maestro- Schrodinger. These selected parameters are suggested to manipulate ligand absorption and cell permeation, distribution, metabolism, excretion, and toxicity. As shown in Additional file 1: Table S1, only compound **2i** and **2h** revealed a little bit of deviation from the recommended PISA and QPlogS parameter ranges that are supposed to be compensated by other recorded ideal parameters. Otherwise, other molecules concerning all applied ADME-T analysis displayed ADME-T values within the ideal ranges, so all ligands are highly considered safe for future drugs.

## Conclusion

The synthesized compounds showed potent inhibitory activity against COX-1 and COX-2 enzymes. However, most compounds have COX-2 inhibition selectivity. The results showed a promising group of compounds having a thiazole moiety. The cytotoxic evaluation results revealed that almost all of the evaluated compounds have no cytotoxic activities in different cell lines except for compound **2f** with moderate activities. The COX enzyme effective doses were at least ten times lower than the cytotoxic concentrations. Molecular docking studies showed that the **2d, 2e, 2f,** and **2i** molecules were able to set optimally and interact ideally within the COX-2 binding site if compared to COX-1. However, they could not interact with key residue ARG-513 of the COX-2 enzyme, and thus could not open the polar secondary binding pocket. They successfully formed hydrogen bonds with the other surrounding residues such as ARG-120, HIS-90, and TYR-355, hydrophobic interactions, and pi-pi-interactions that participated mainly in boosting affinity. The existence of large groups like the three methoxy groups substituted to the phenyl ring, like in the **2f** molecule, led to generating steric clashes and losing pi-stacking or pi-cation interactions within the COX-1 binding site, which resulted in dimensioning affinity. On the other hand, these negative aspects were bypassed and that large group contributed positively to boosting COX-2 activity via optimally occupying the available binding pocket and localizing the molecule ideally within the COX-2 binding site. The proposed affinities calculated using Prime MM-GBSA were in agreement with the recorded XP-glide docking scores and the observed biological activity. Furthermore, the density functional theory calculations were confirmed for a small amount of the HOMO–LUMO energy gap, indicating high chemical reactivity. All ligand molecules show admissible properties of the ADME and are considered potential drug candidates for prospective research. The data achieved in this research will have a valuable contribution to the perspective plans that should aim to design and synthesize more analogs with bulky groups on the phenyl ring to increase the selectivity of the COX-2 enzyme, like the most selective compound, **2f**.

## Supplementary Information

**Additional file 1.** contains the IUPAC name, chemical structures, NMR spectrums, free energy data, Crystal binding mode and ADME-T properities of the synthesized molecules.

## Data Availability

All data generated or analysed during this study are included in this published article (and its Additional information files).

## Abbreviations

COX: Cyclooxgenase enzyme
NMR: Nuclear magnetic resonance
M.P.: Melting points
HRMS: High-resolution mass spectrometer
DMSO: Dimethyl sulfoxide
IC_50_: Half maximal inhibitory concentration
MCF7: Human breast cancer cell line
Conc.: Concentration
