# Supplementary material for: Design, synthesis, molecular docking studies and biological evaluation of thiazole carboxamide derivatives as COX inhibitors
Source: BMC Chem. 2023 Mar 6;17(1):11. doi: 10.1186/s13065-023-00924-3 (PMC9987136; doi:10.1186/s13065-023-00924-3)
Supplement: Supplementary file 1 — Additional file 1. contains the IUPAC name, chemical structures, NMR spectrums, free energy data, Crystal binding mode and ADME-T properities of the synthesized molecules. [file 13065_2023_924_MOESM1_ESM.docx]

**Supplementary Material**

**Design, Synthesis, Molecular docking studies and biological evaluation of thiazole-carboxamide derivatives as COX inhibitors**

**Mohammed Hawash^a,*^, Nidal Jaradat^a^, Murad Abualhasan^a^,** **Murat Kadır Şüküroğlu^b^, Mohammed T. Qaoud^b^, Deniz Cansen Kahraman^c^, Heba daraghmeh^a^, Leen Maslamani^a^, Mais Sawafta^a^, Ala Ratrout^a^, Linda Issa^a^**

^a^ Department of Pharmacy, Faculty of Medicine and Health Sciences, An-Najah National University, Nablus, Palestine.

^b^ Department of Pharmaceutical Chemistry, Faculty of Pharmacy, Gazi University, Ankara, Turkey.

^c^ Cancer Systems Biology Laboratory, Graduate School of Informatics, Middle East Technical University, 06800, Ankara, Turkey

^*^**Corresponding author**: **Mohammed Hawash**, Department of Pharmacy, Faculty of Medicine and Health Sciences, An-Najah National University, Nablus, PALESTINE, orcid.org/0000-0001-5640-9700; Phone: +972569939939; Email: mohawash@najah.edu.

| **Figures and Tables** | **Pages** |
| --- | --- |
| **Figure S1. Figure S1. 2-(4-methoxyphenyl)-N-phenylthiazole-4-carboxamide *(2a) LMH-3………………………………..*** | **4** |
| **Figure S2. N-(3,4-dimethoxyphenyl)-2-(4-methoxyphenyl)thiazole-4-carboxamide *(2b)* LMH-1………………………….** | **6** |
| **Figure S3. N-(3,5-dimethoxyphenyl)-2-(4-methoxyphenyl)thiazole-4-carboxamide *(2c) LMH-4………………………….*** | **8** |
| **Figure S4. N-(2,5-dimethoxyphenyl)-2-(4-methoxyphenyl)thiazole-4-carboxamide *(2d) LMH-7………………………….*** | **10** |
| **Figure S5. N-(2,4-dimethoxyphenyl)-2-(4-methoxyphenyl)thiazole-4-carboxamide *(2e) LMH-9………………………….*** | **12** |
| **Figure S6. 2-(4-methoxyphenyl)-N-(3,4,5- trimethoxyphenyl)thiazole-4-carboxamide *(2f) LMH-2……………………….*** | **14** |
| **Figure S7. N-(4-chloro-2,5-dimethoxyphenyl)-2-(4-methoxyphenyl)thiazole-4-carboxamide *(2g) LMH-5………………..*** | **16** |
| **Figure S8. N-(4-(tert-butyl)phenyl)-2-(4-methoxyphenyl)thiazole-4-carboxamide *(2h) LMH-6…………………………..*** | **18** |
| **Figure S9. 2-(4-methoxyphenyl)-N-(4-(thiophen-2-yl)phenyl)thiazole-4-carboxamide *(2i) LMH-8………………………*** | **20** |
| **Equations of Free energy calculations………………………………………………………………………………………..** | **22** |
| **Figure S10. Crystal binding mode of celecoxib, and predicted binding orientations, visualized in COX-1 active site……** | **23** |
| **Figure S11. Crystal binding mode of 2d, and predicted binding orientations, visualized in COX-1 active site………….** | **24** |
| **Figure S12. Crystal binding mode of 2e, and predicted binding orientations, visualized in COX-1 active site…………...** | **25** |
| **Figure S13. Crystal binding mode of 2f, and predicted binding orientations, visualized in COX-1 active site……………** | **26** |
| **Figure S14. Crystal binding mode of 2i, and predicted binding orientations, visualized in COX-1 active site……………** | **27** |
| **Figure S15. Crystal binding mode of celecoxib, and predicted binding orientations, visualized in COX-2 active site……** | **28** |
| **Figure S16. Crystal binding mode of 2d, and predicted binding orientations, visualized in COX-2 active site………….** | **29** |
| **Figure S17. Crystal binding mode of 2e, and predicted binding orientations, visualized in COX-2 active site…………..** | **30** |
| **Figure S18. Crystal binding mode of 2f, and predicted binding orientations, visualized in COX-2 active site…………..** | **31** |
| **Figure S19. Crystal binding mode of 2i, and predicted binding orientations, visualized in COX-2 active site…………..** | **32** |
| **Figure S20: Plots of highest occupied molecular orbital (HOMO) and lowest unoccupied molecular orbital (LUMO) of compounds 2a, 2b, 2c, 2g and** | **33** |
| **Table S1: The ADME-T properties of synthesized molecules…………………………………………………………………** | **34** |

**Figure S1. 2-(4-methoxyphenyl)-N-phenylthiazole-4-carboxamide *(2a) LMH-3***

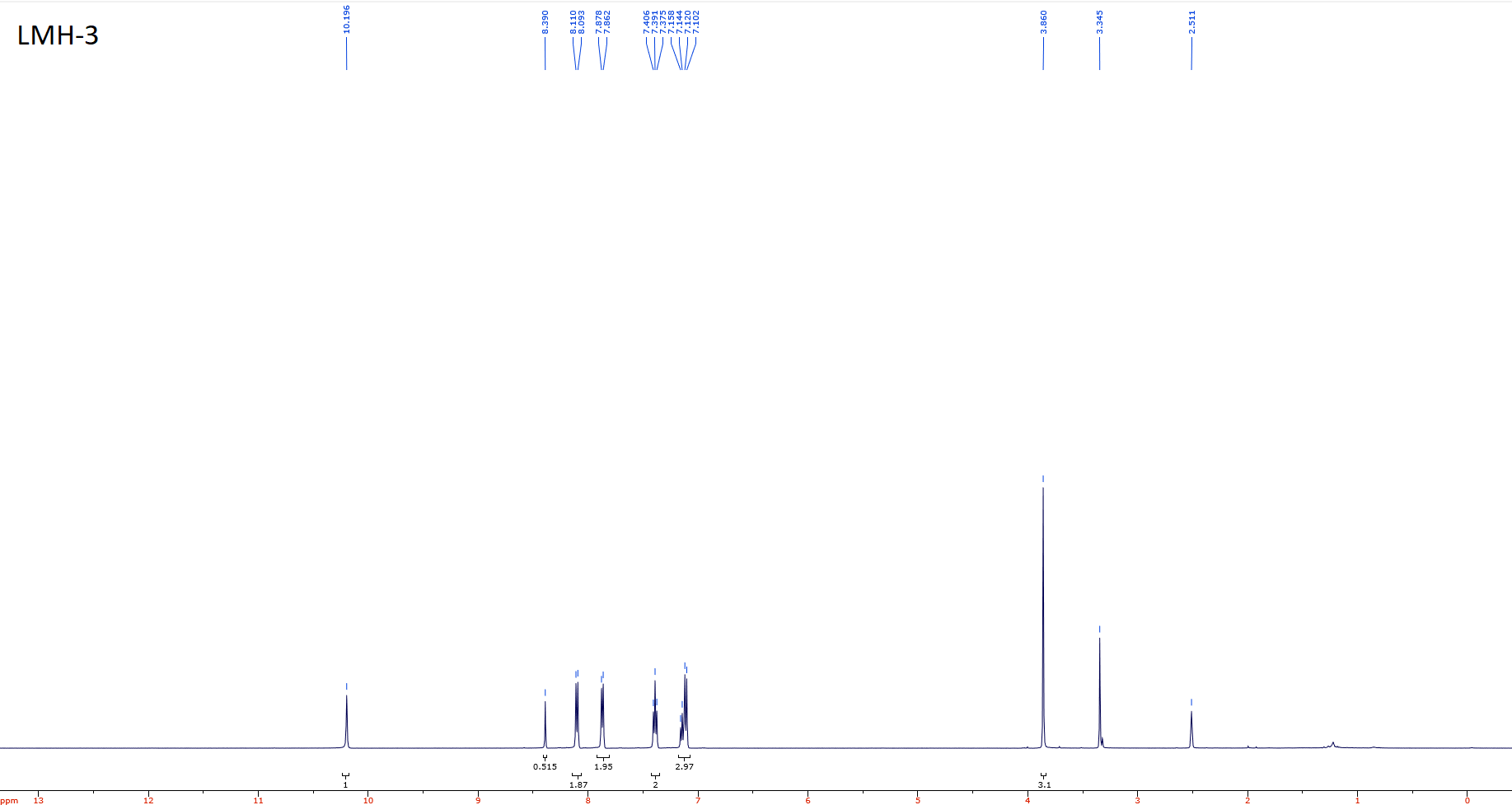


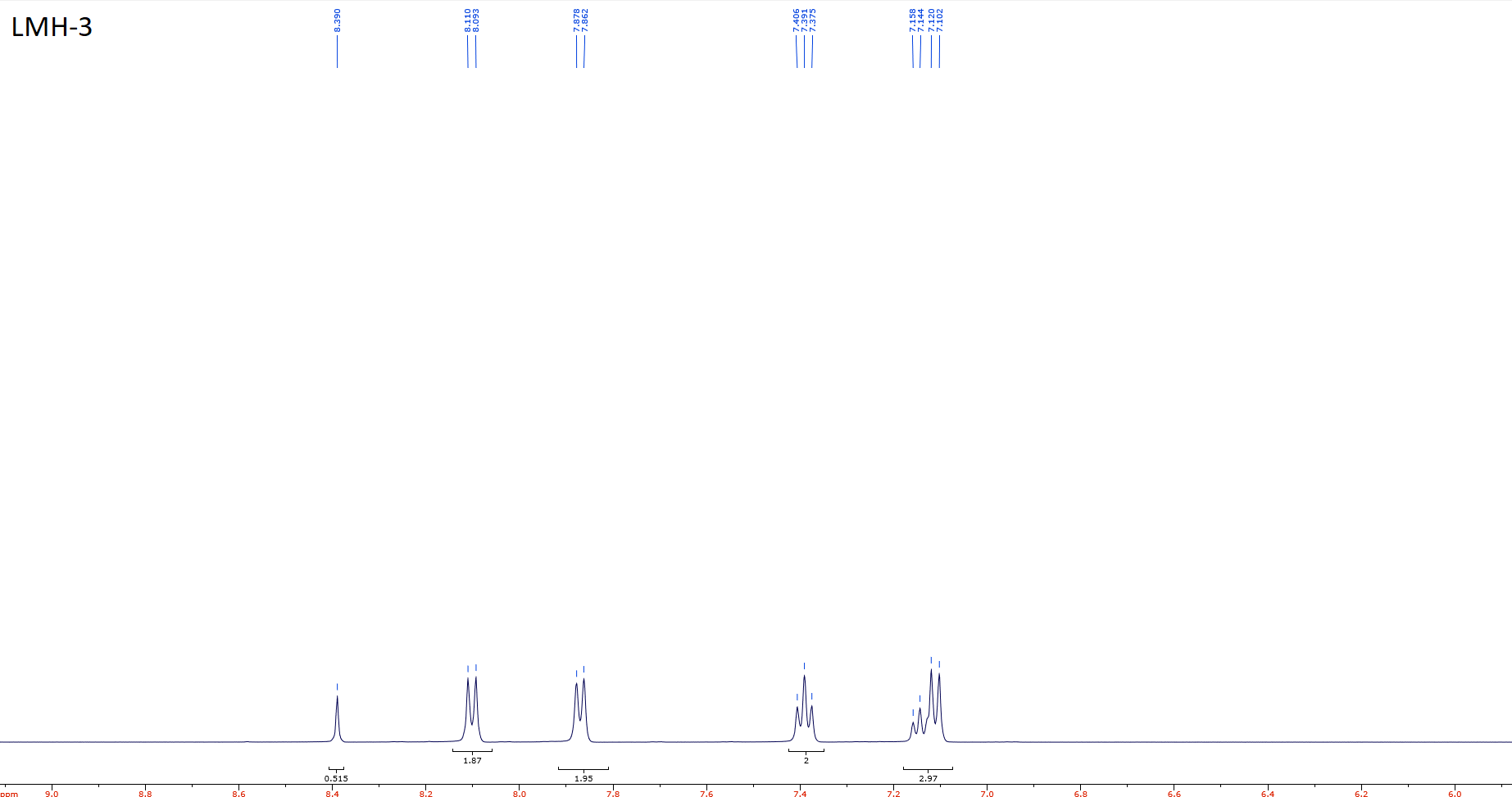


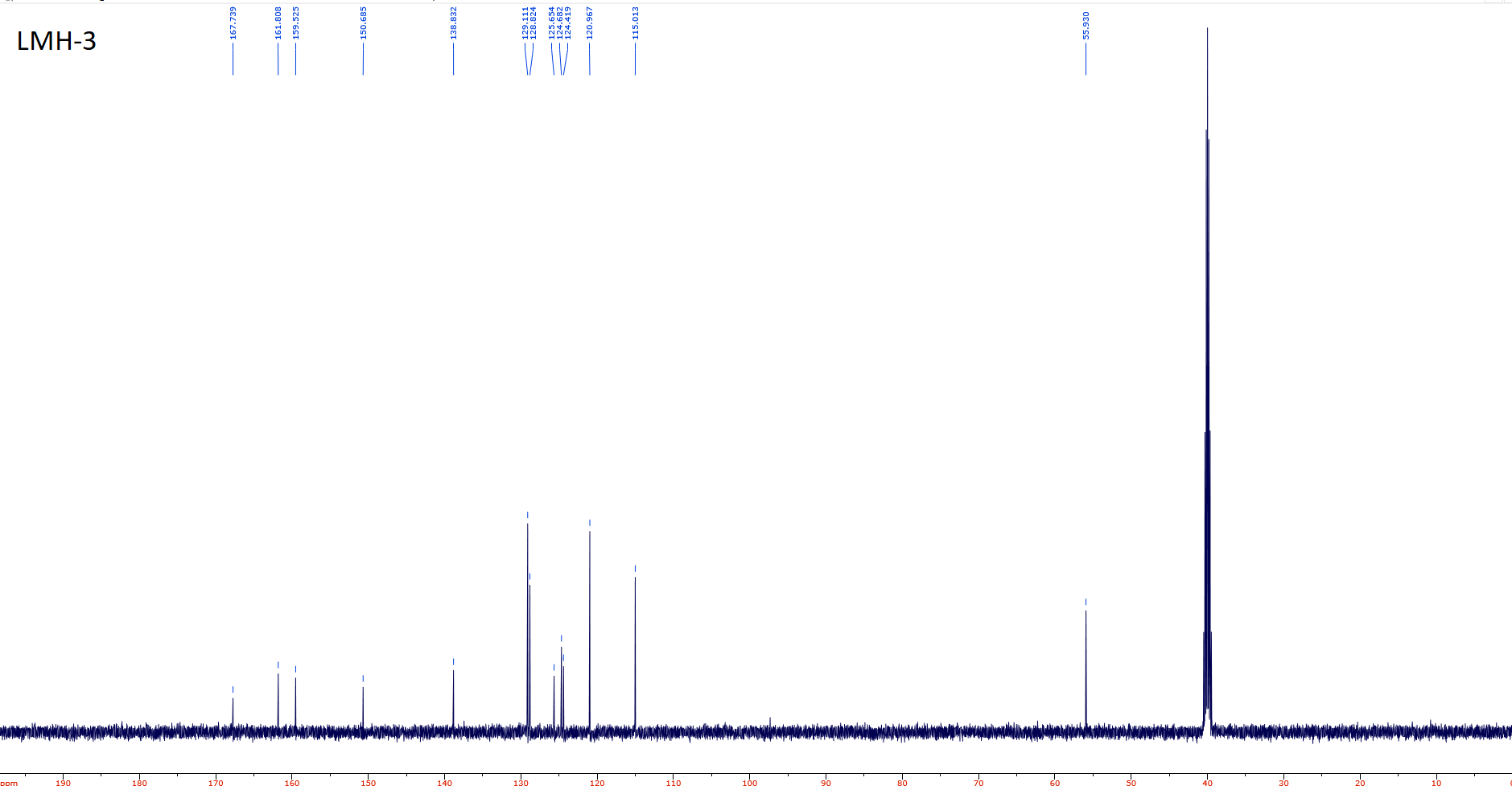


**Figure S2. N-(3,4-dimethoxyphenyl)-2-(4-methoxyphenyl)thiazole-4-carboxamide *(2b)* LMH-1**

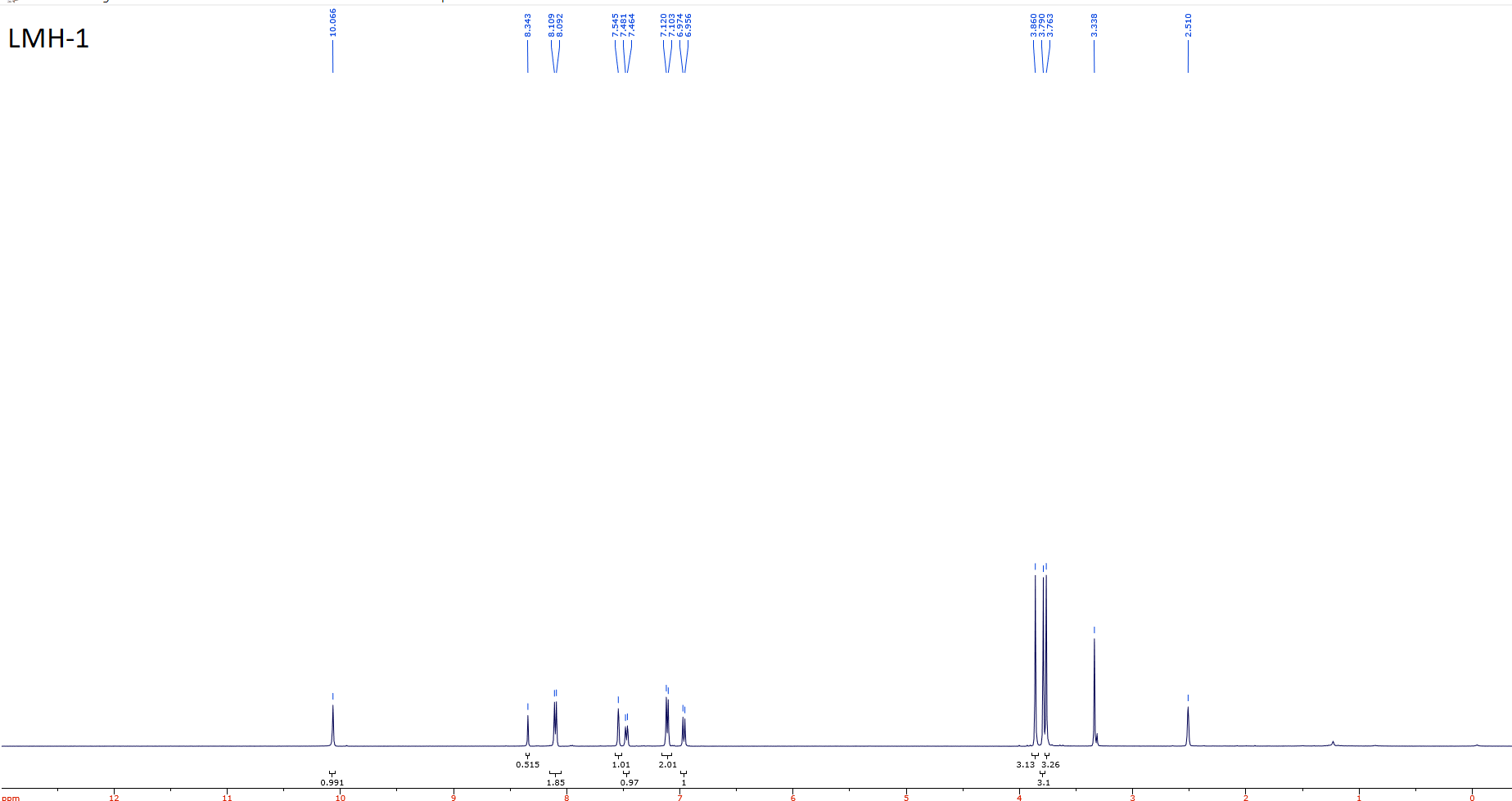


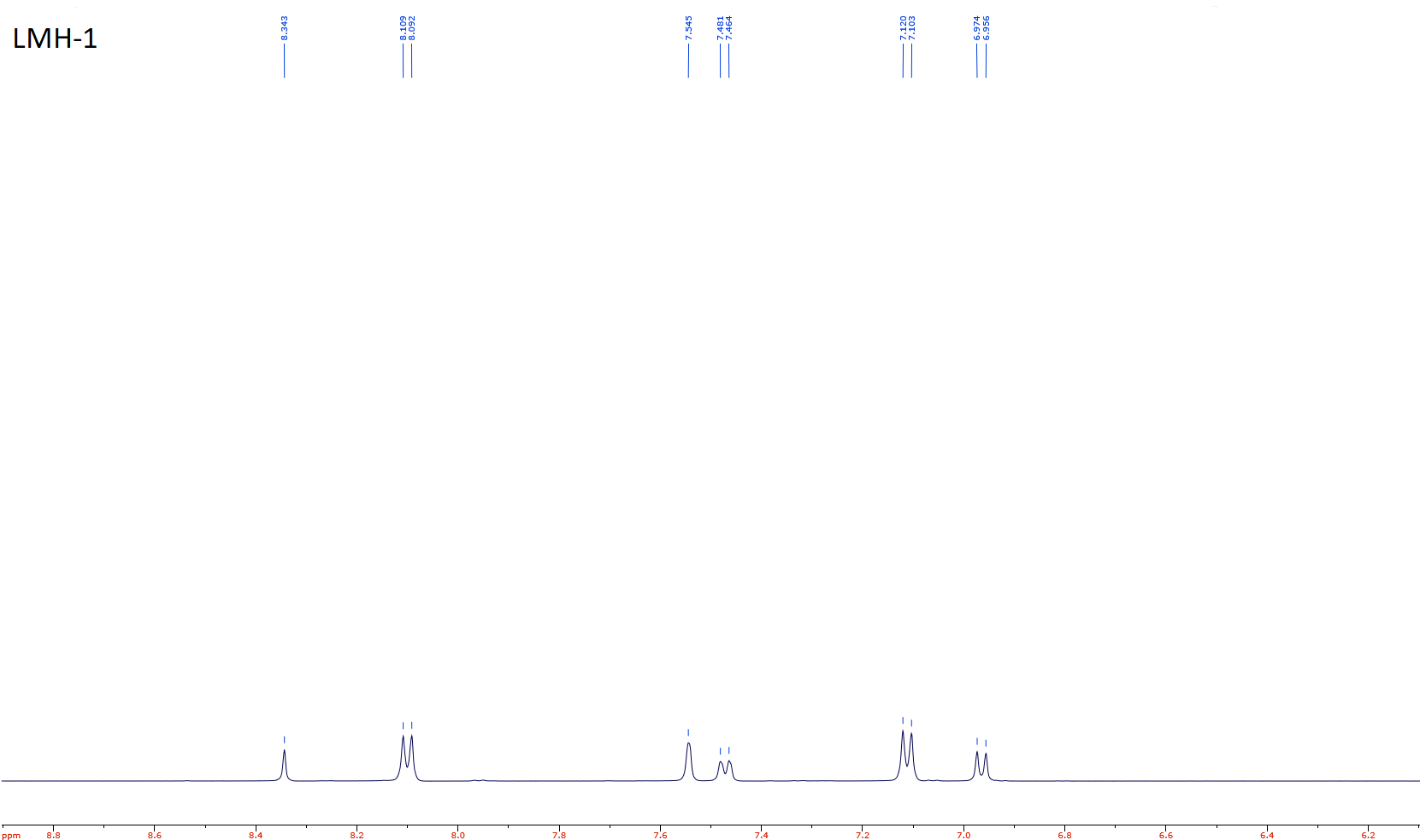


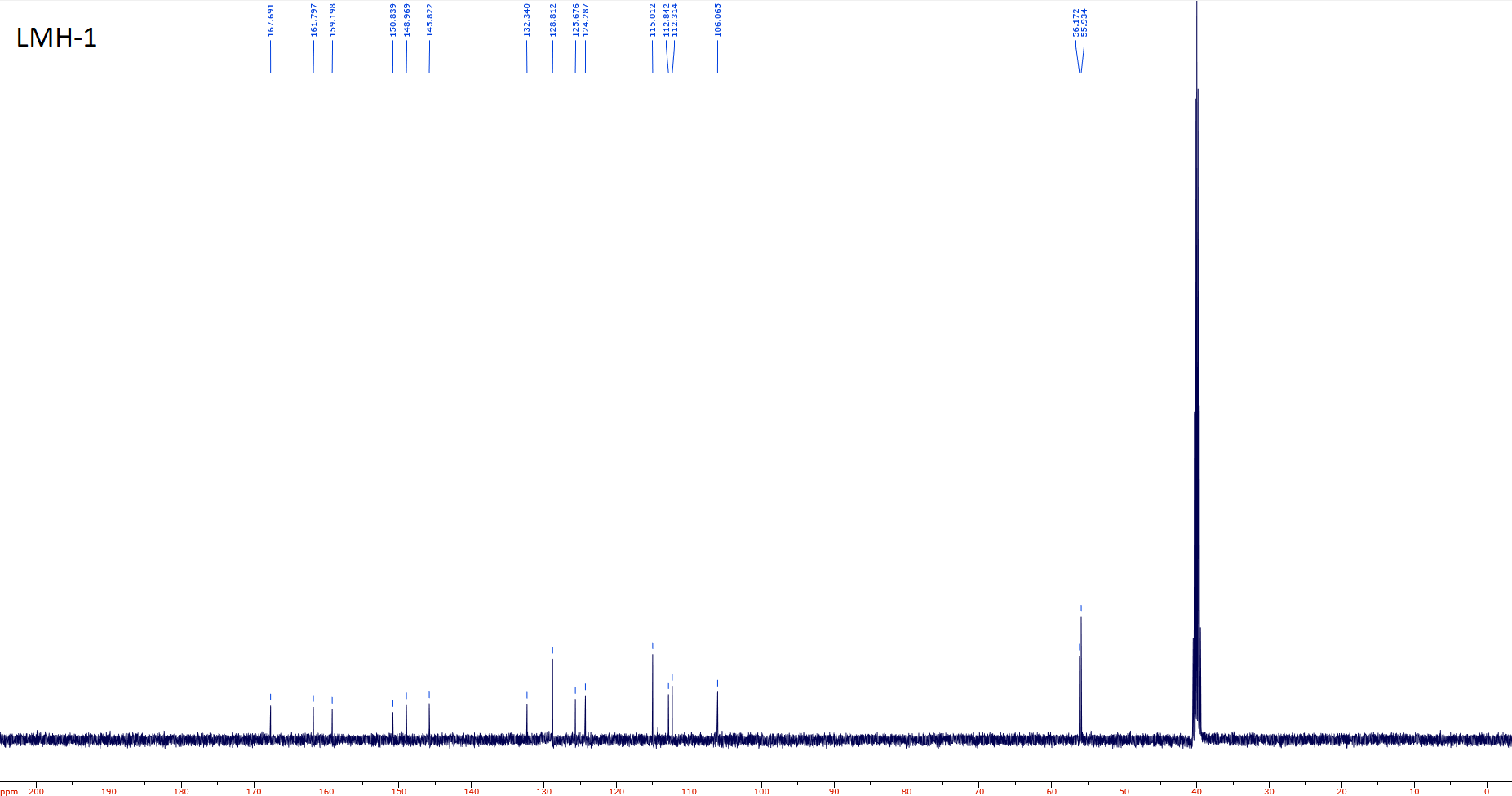


**Figure S3. N-(3,5-dimethoxyphenyl)-2-(4-methoxyphenyl)thiazole-4-carboxamide *(2c) LMH-4***

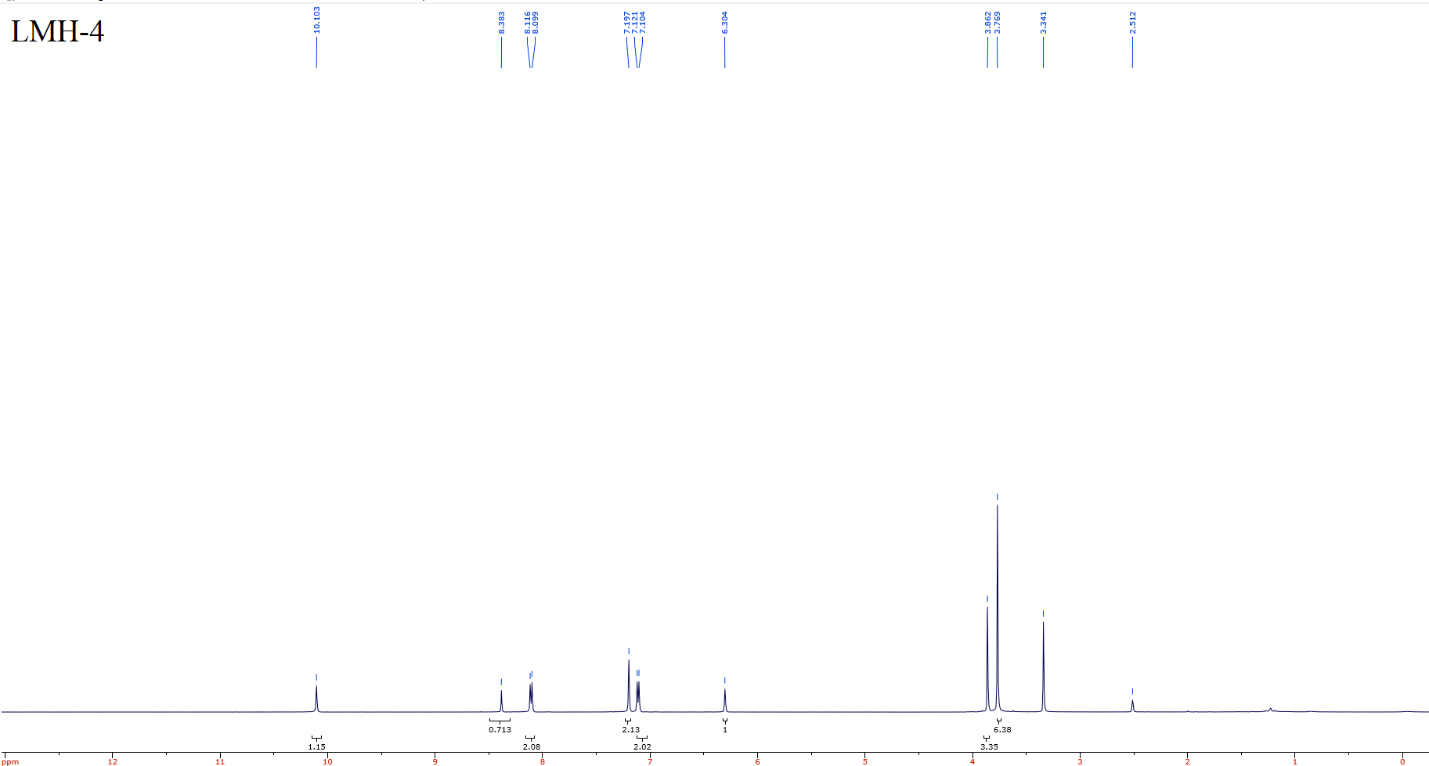


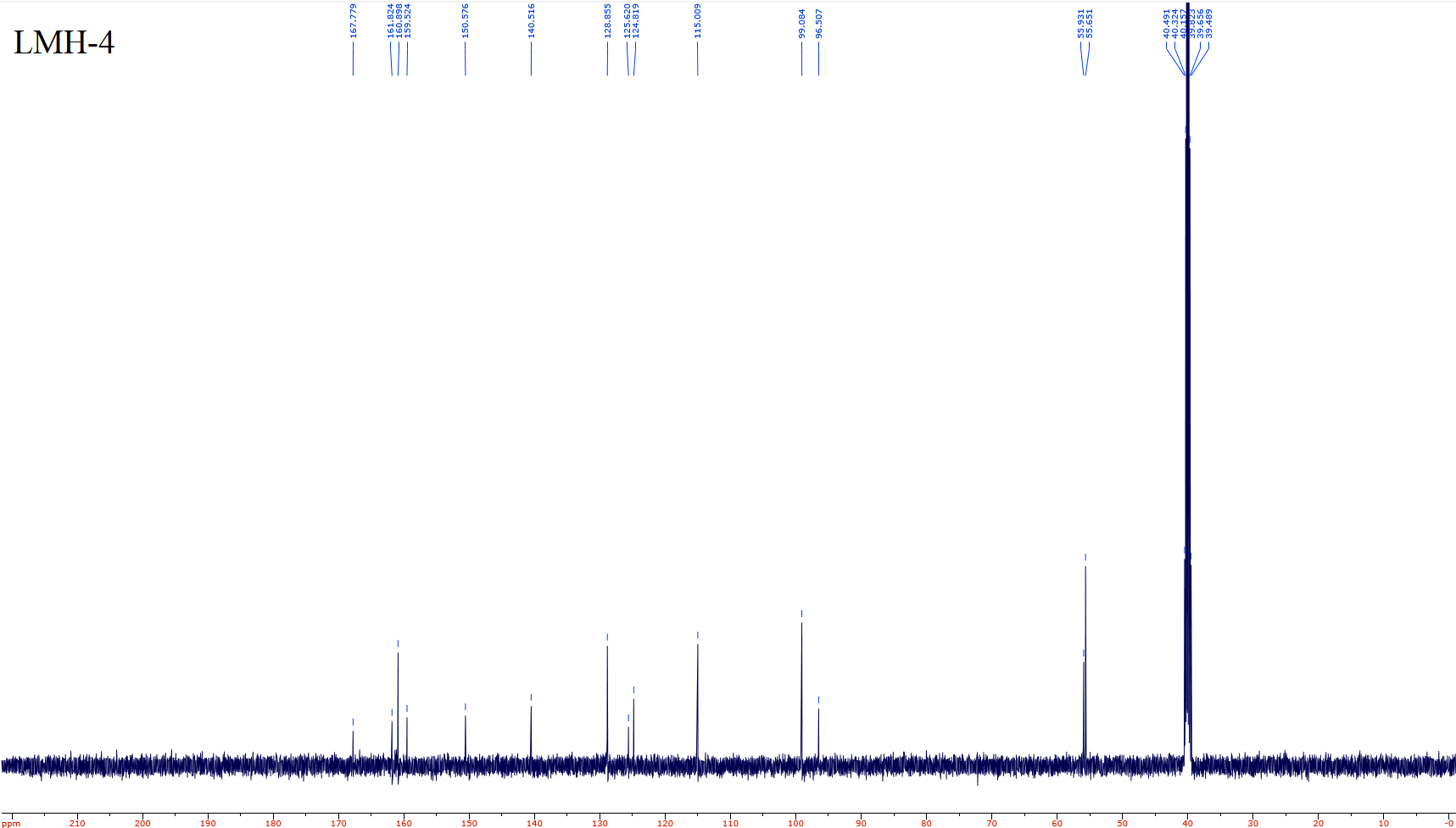


**Figure S4. N-(2,5-dimethoxyphenyl)-2-(4-methoxyphenyl)thiazole-4-carboxamide *(2d) LMH-7***

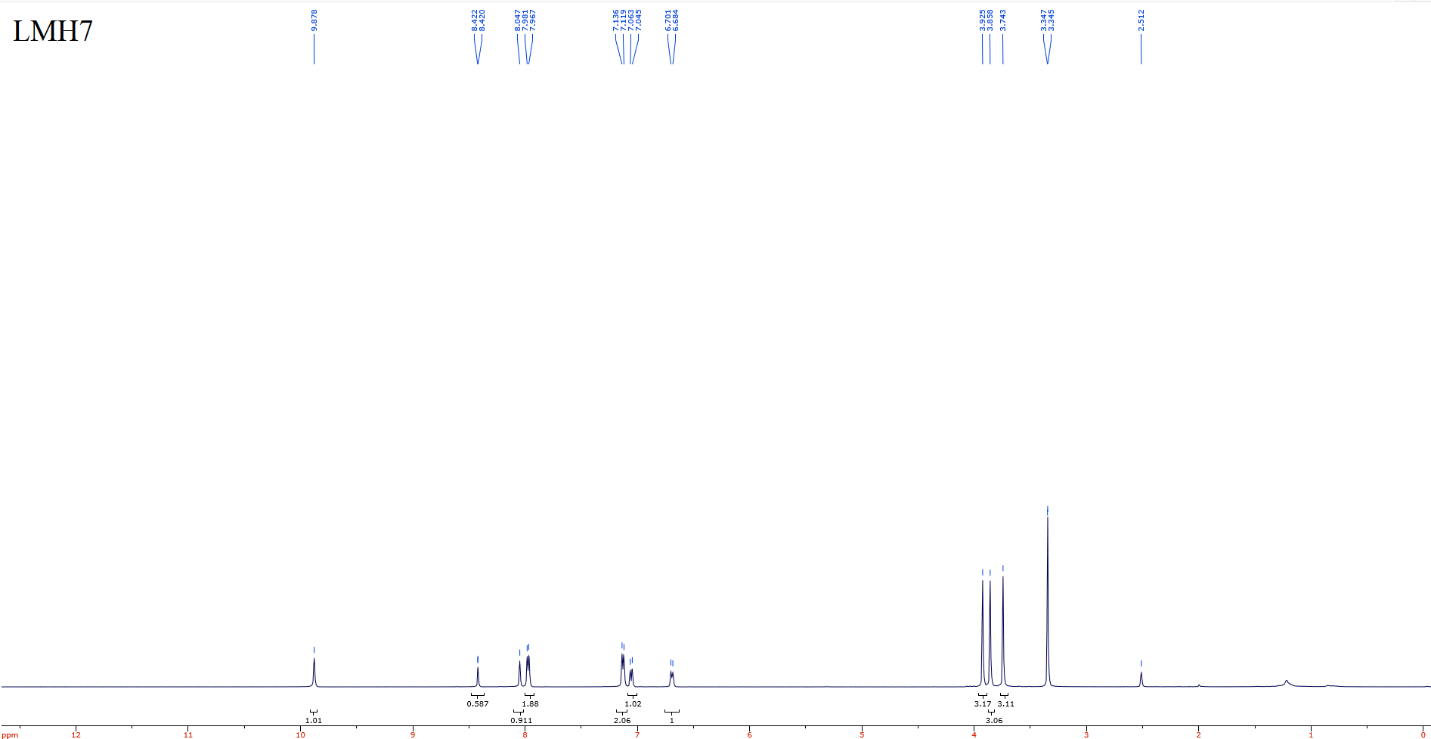


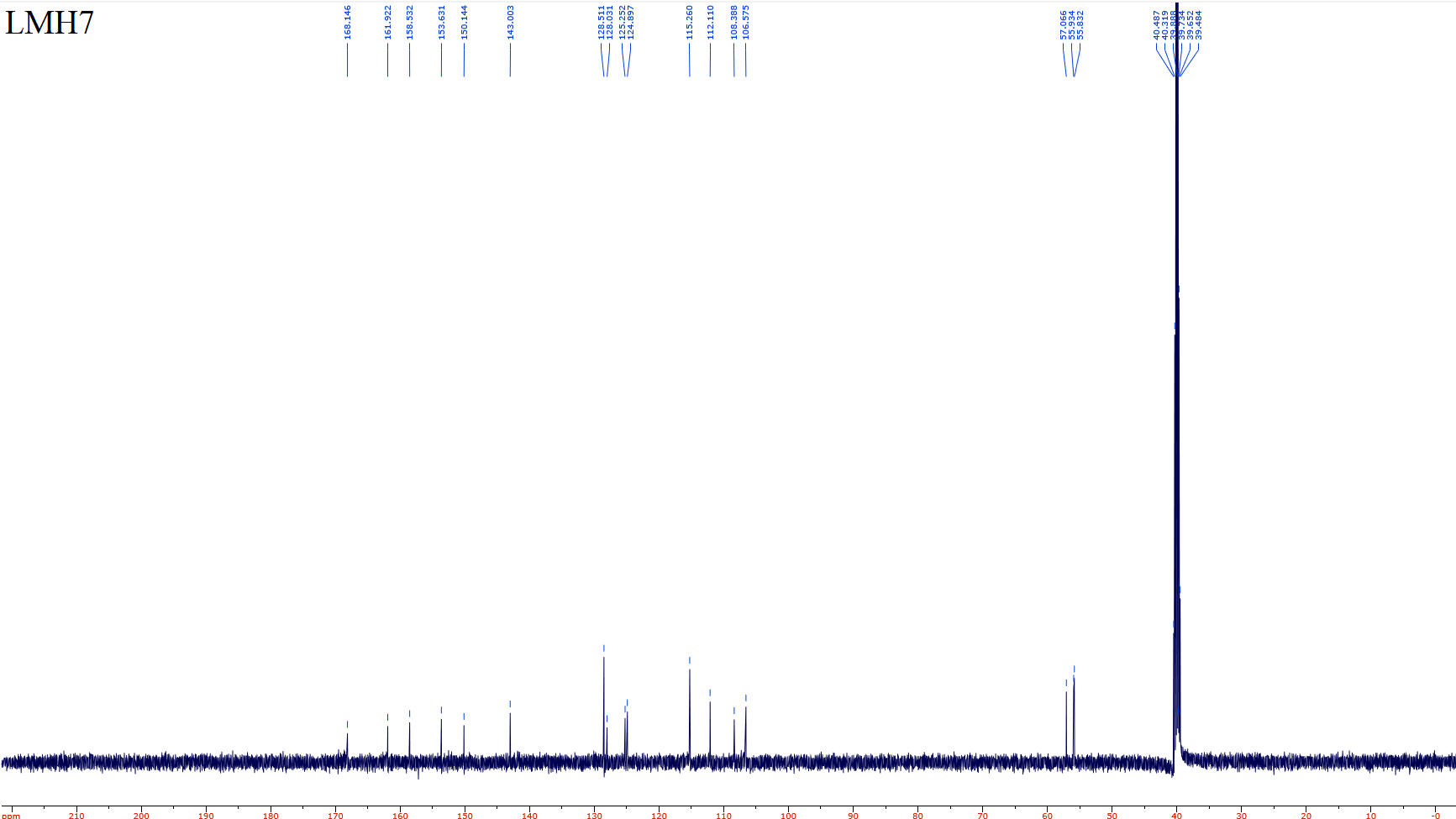


**Figure S5. N-(2,4-dimethoxyphenyl)-2-(4-methoxyphenyl)thiazole-4-carboxamide *(2e) LMH-9***

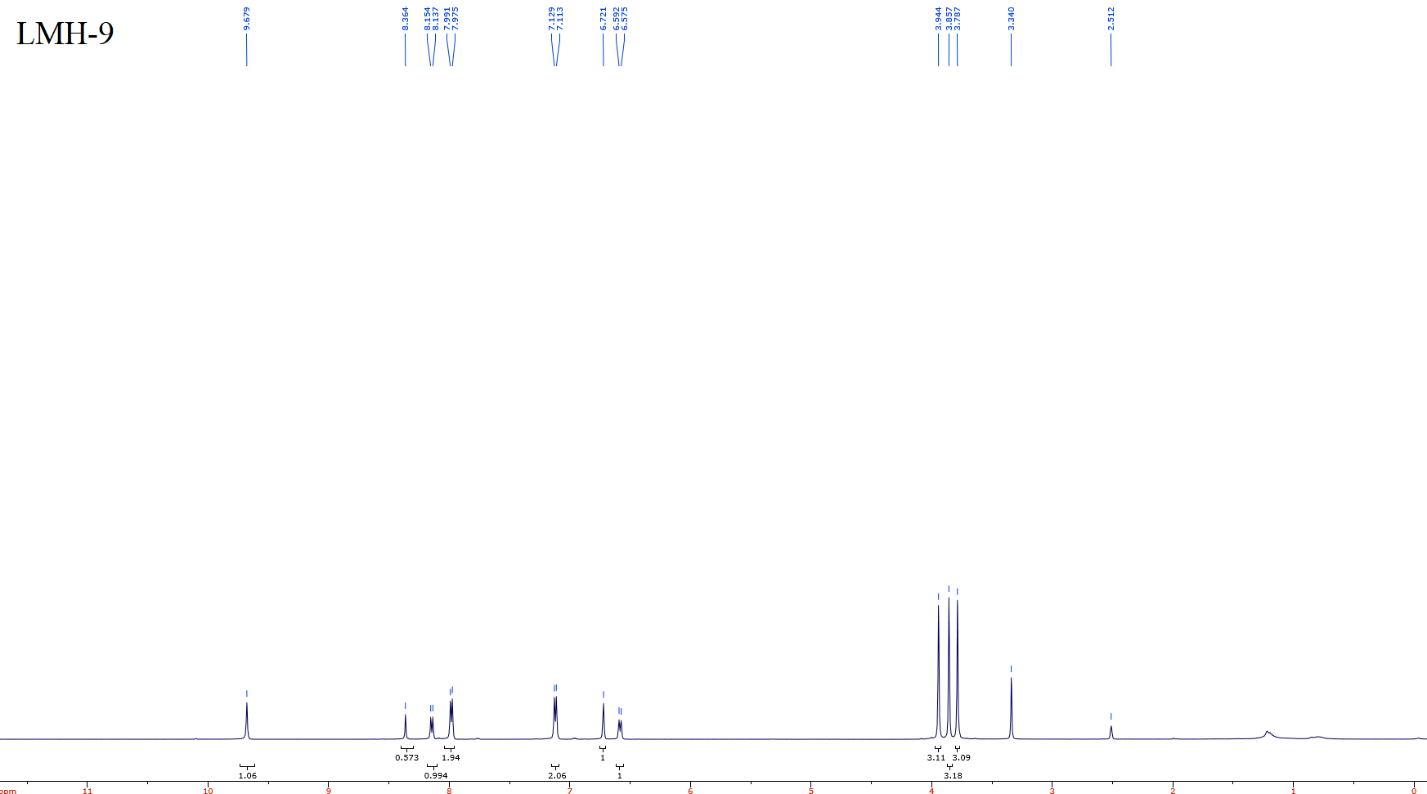


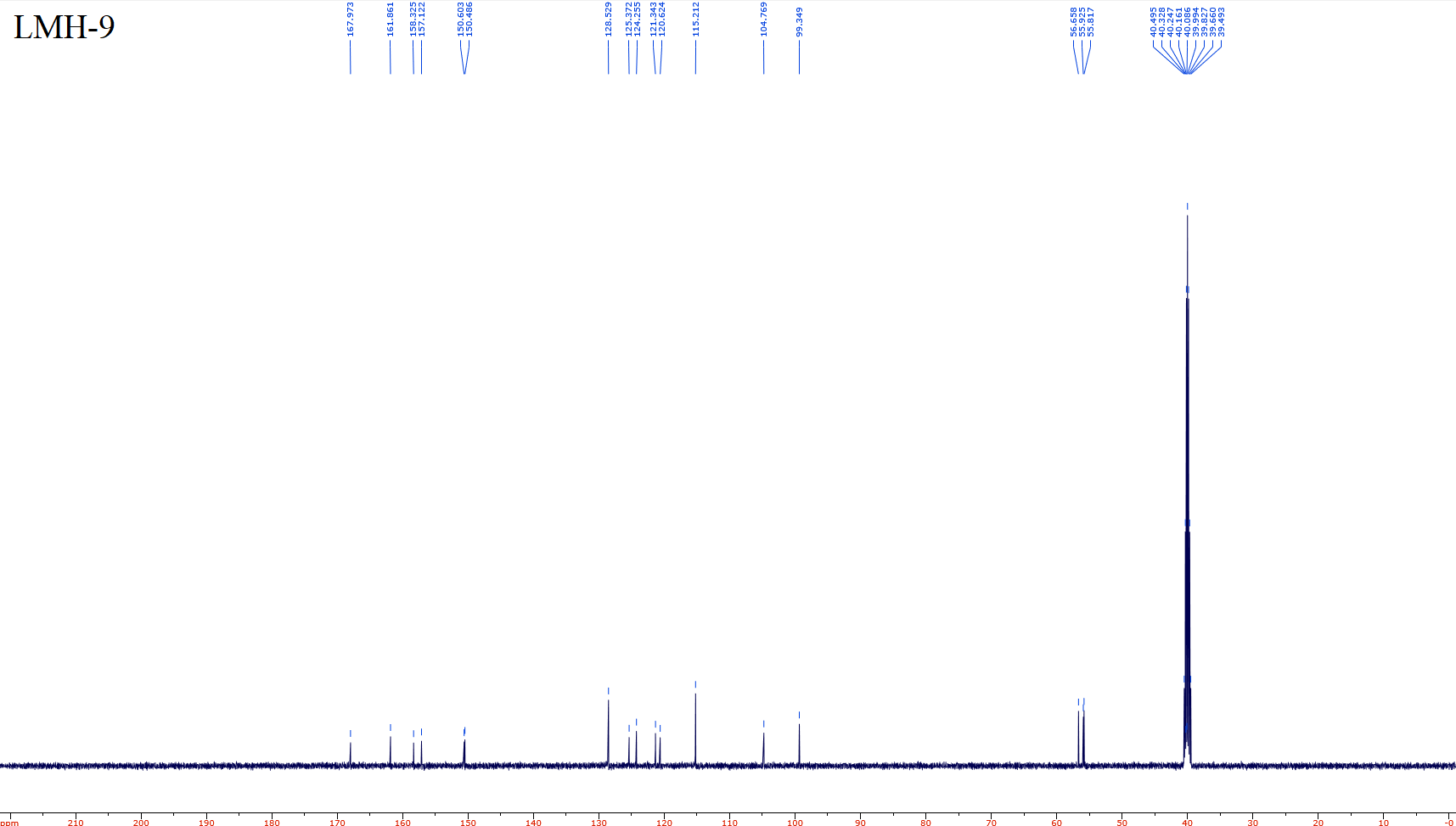


**Figure S6. 2-(4-methoxyphenyl)-N-(3,4,5- trimethoxyphenyl)thiazole-4-carboxamide *(2f) LMH-2***

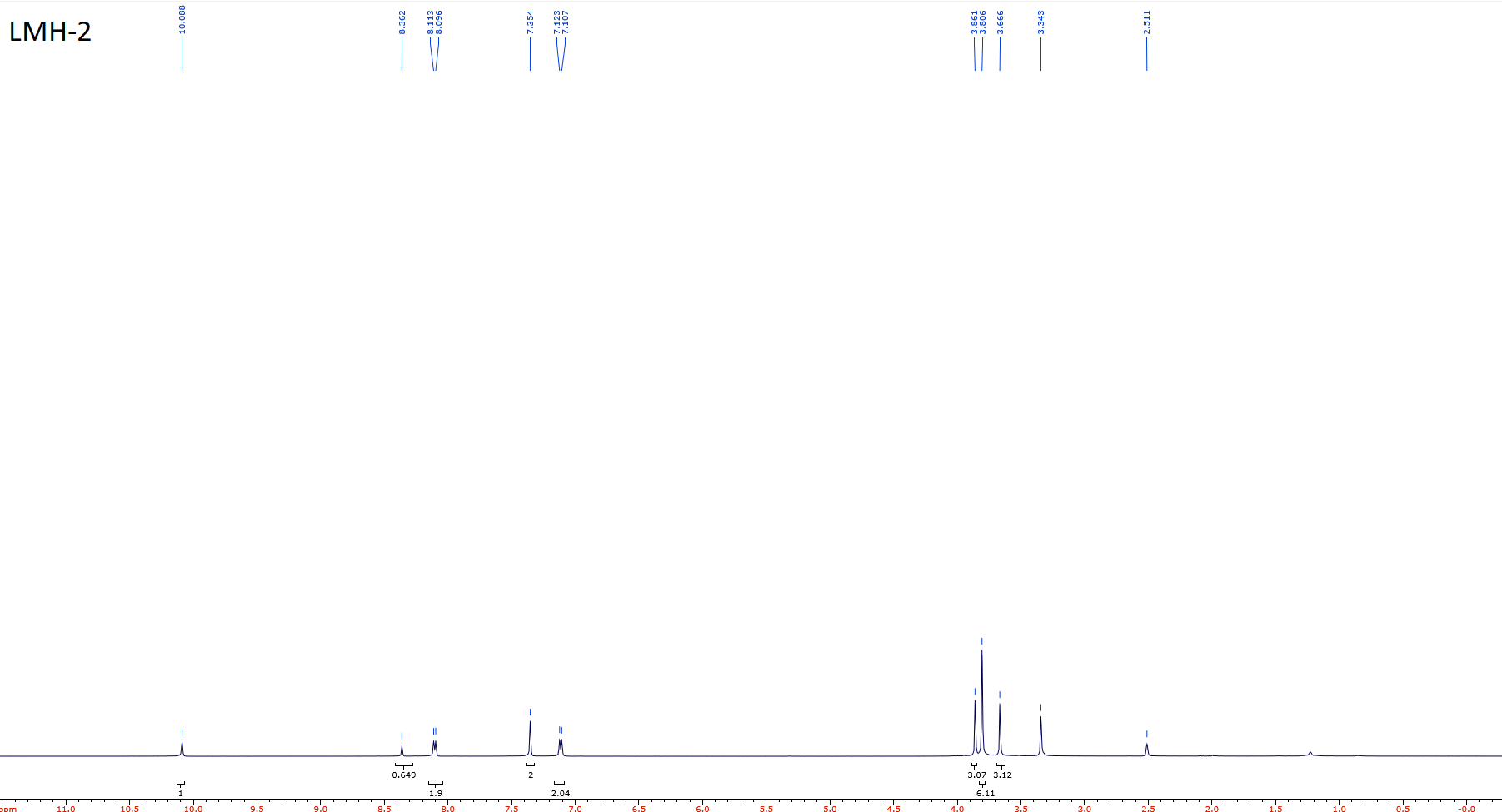


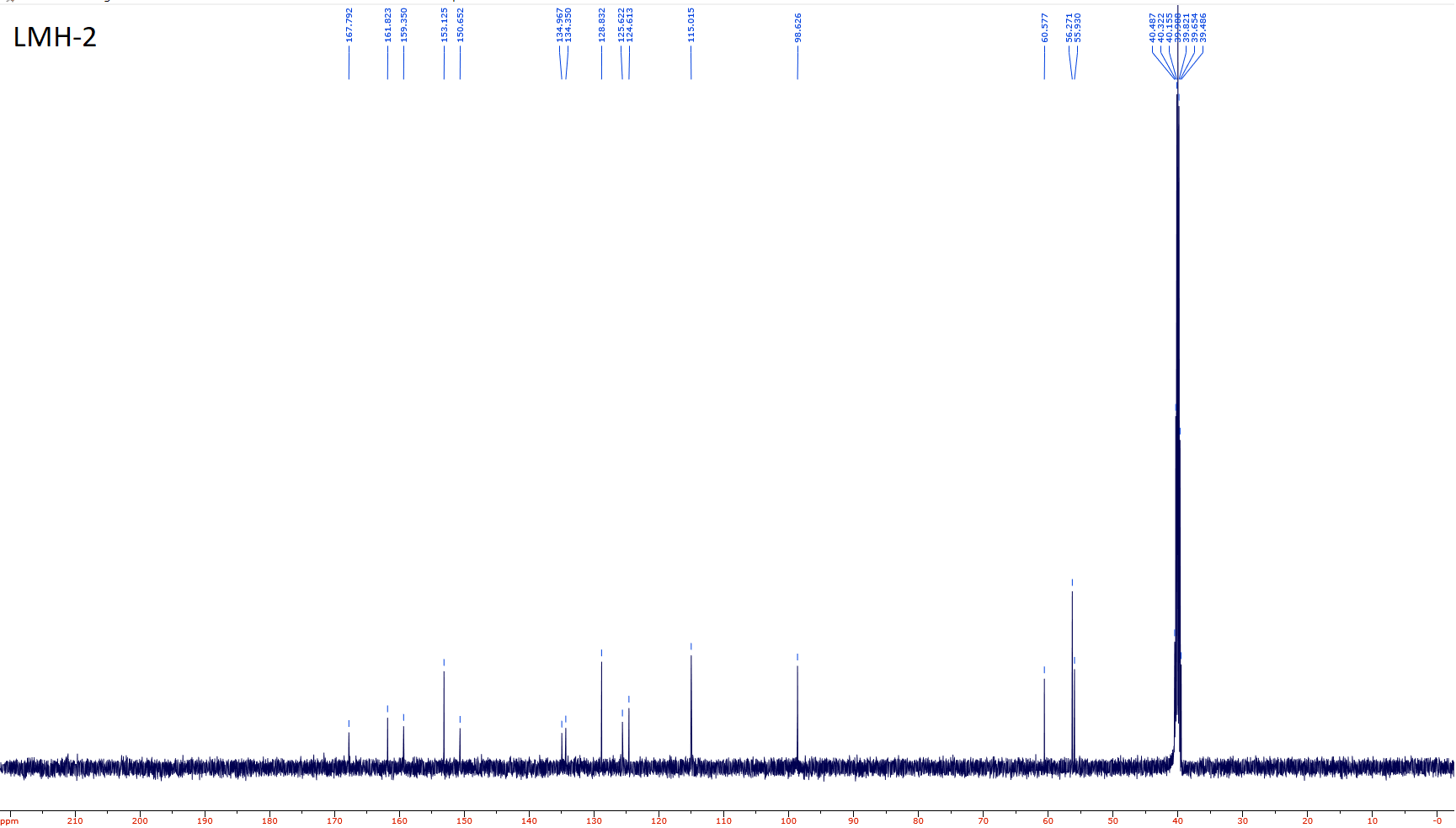


**Figure S7. N-(4-chloro-2,5-dimethoxyphenyl)-2-(4-methoxyphenyl)thiazole-4-carboxamide *(2g) LMH-5***

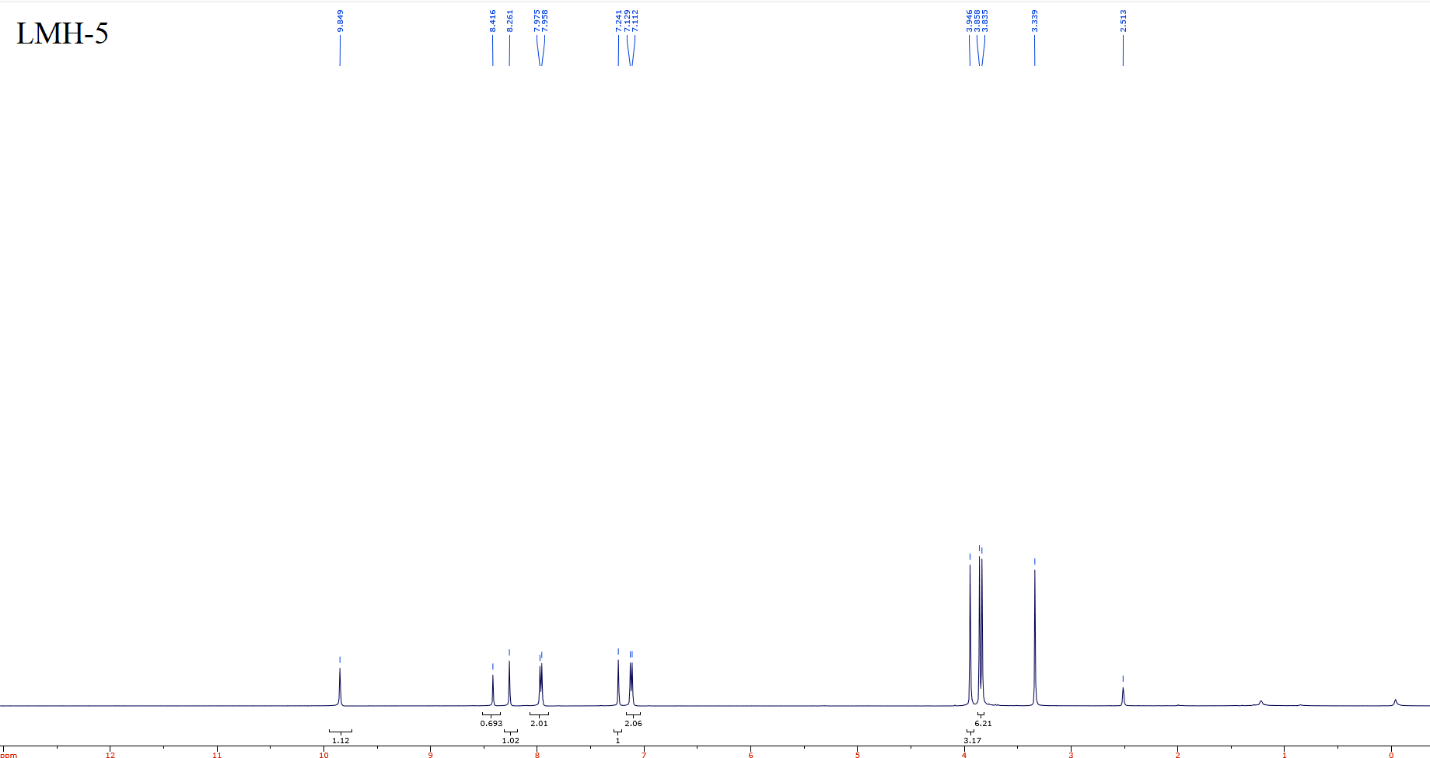


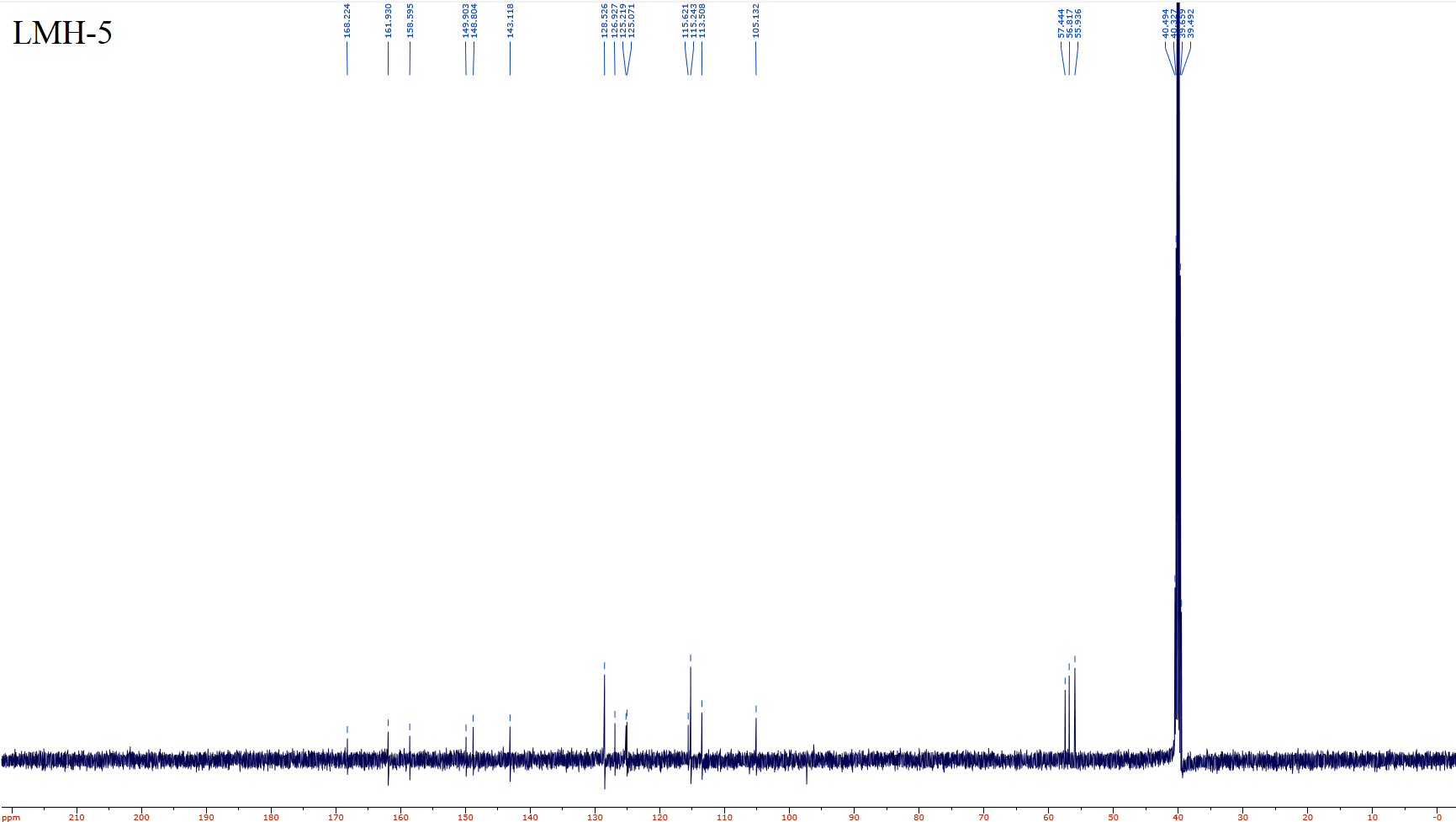


**Figure S8. N-(4-(tert-butyl)phenyl)-2-(4-methoxyphenyl)thiazole-4-carboxamide *(2h) LMH-6***

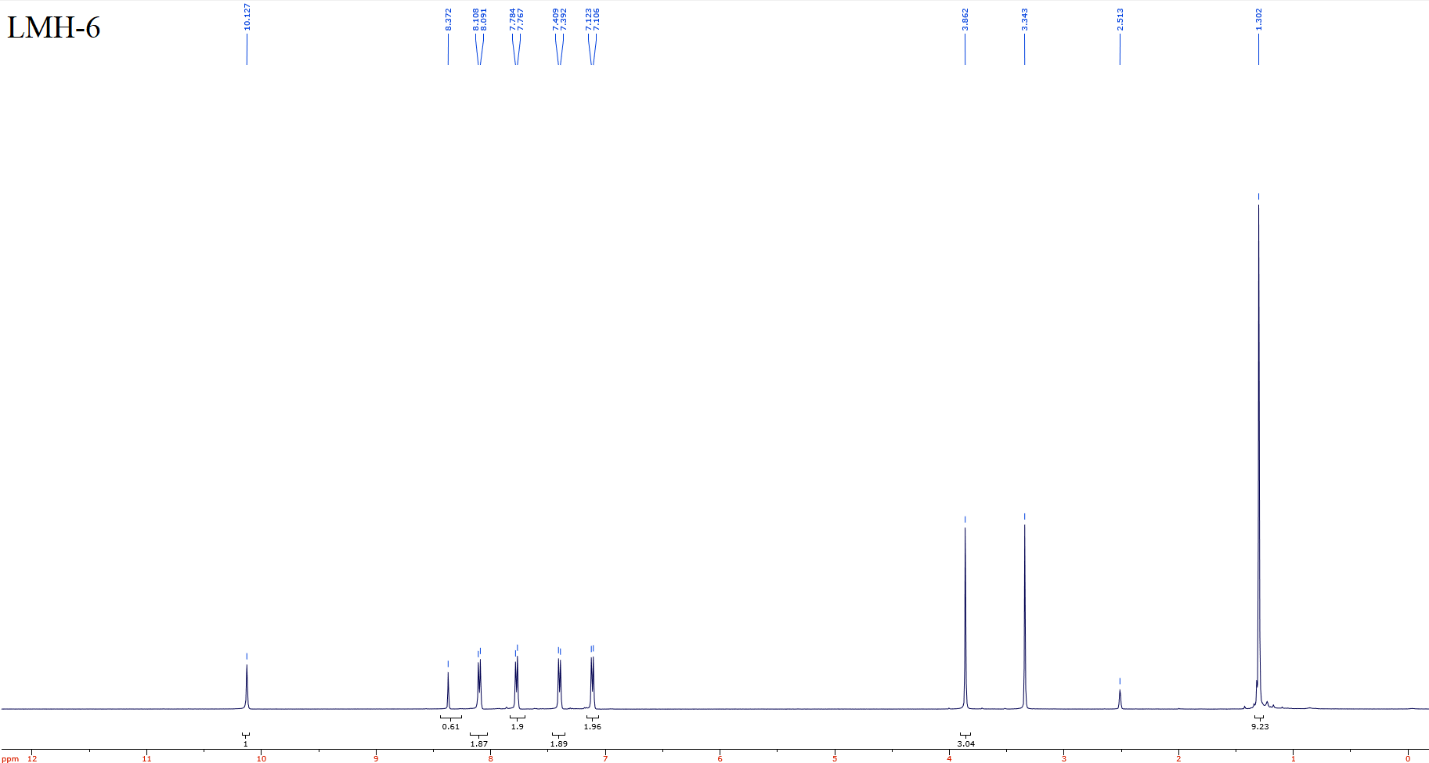


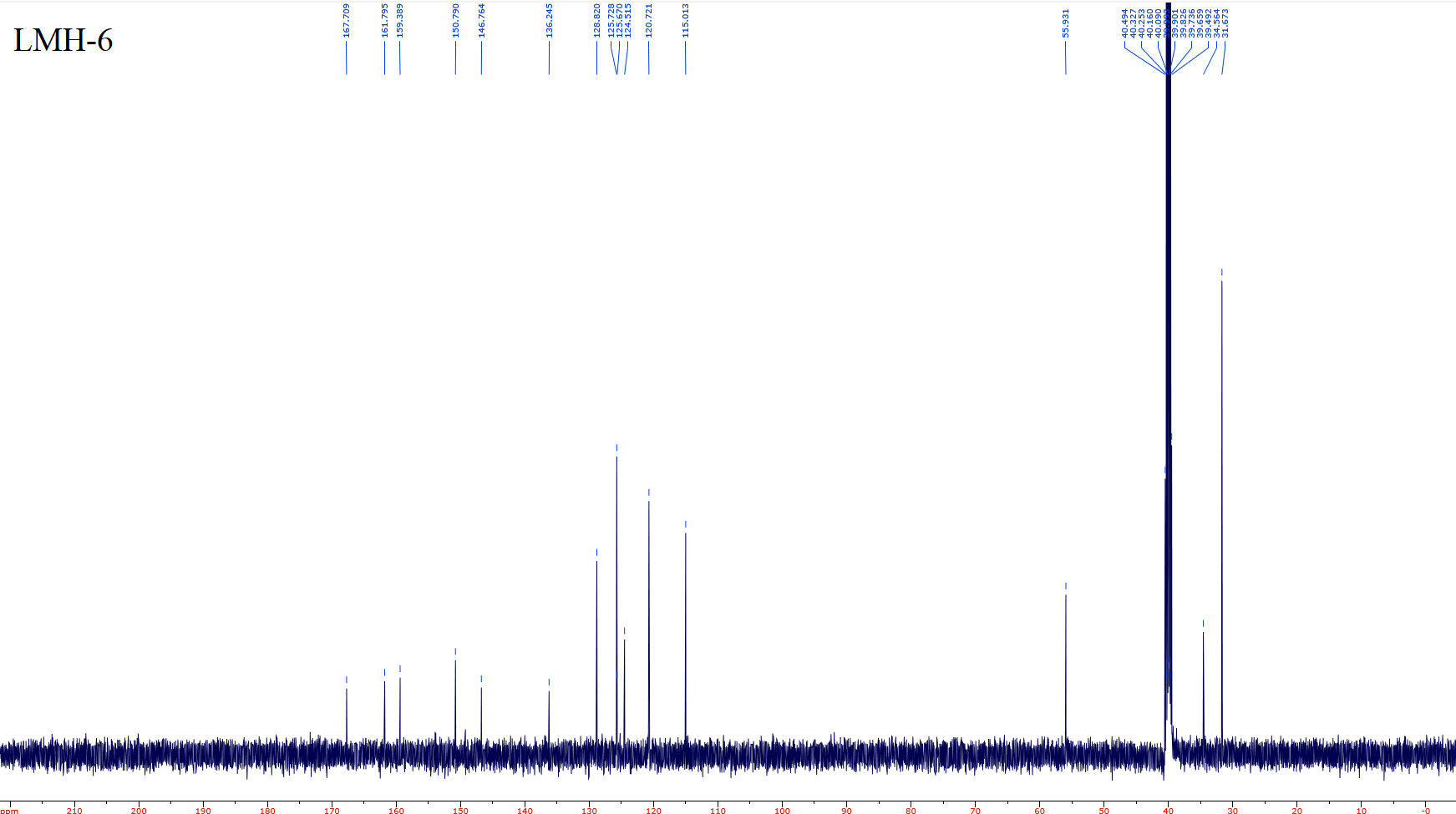


**Figure S9. 2-(4-methoxyphenyl)-N-(4-(thiophen-2-yl)phenyl)thiazole-4-carboxamide *(2i) LMH-8***

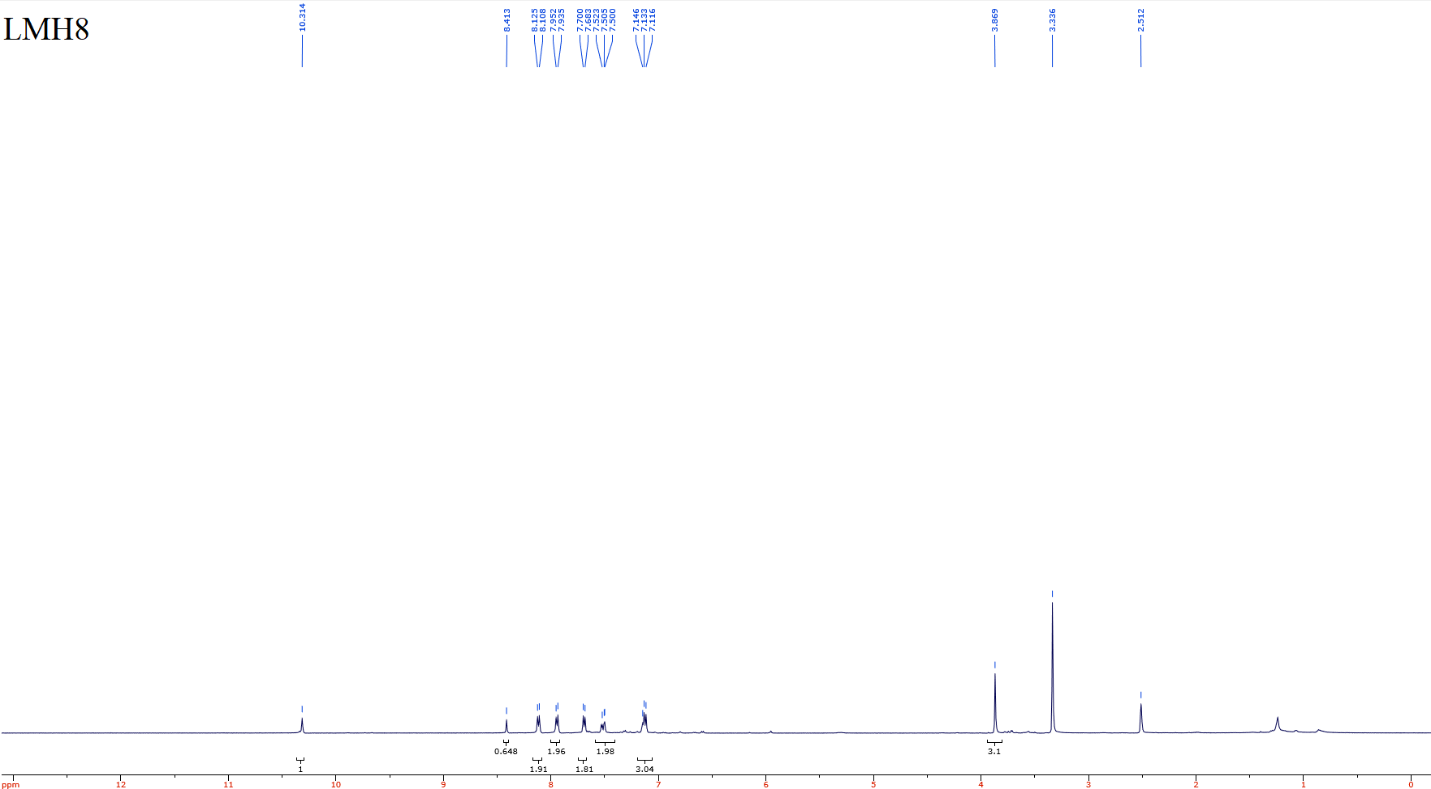


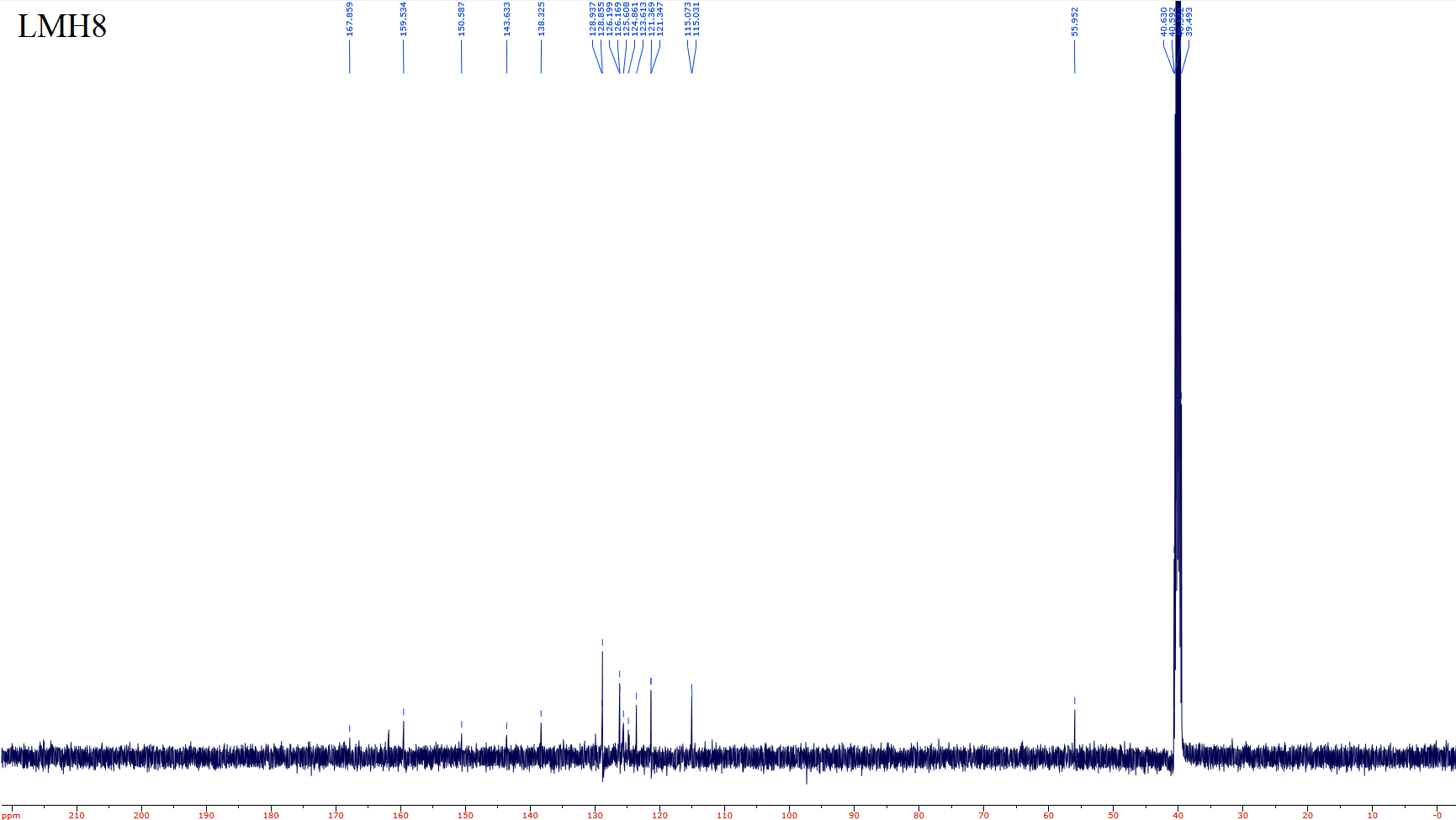


**Free energy calculations’ equations by using Prime MM-GBSA**

ΔG_bind_ = ΔE+ ΔG_SOLV_ + ΔG_SA_  ---------------- Eq.1

Where:

- The minimized energies (ΔE) could be calculated via equation 2

ΔE = E_complex_ – (E_protein_ + E_ligand_) ----------------Eq.2

- E_complex_, E_protein_ , and E_ligand_ are the minimized energies of protein-ligand complex, protein, and ligand, respectively.

- The free energies of salvation (ΔG_SOLV_ ) could be calculated via equation 3

ΔG_SOLV_ = G_solv(complex)_ – (G_solv (protein)_ + G_solv (ligand)_ ----------------Eq.3

G_solv(complex)_, (G_solv (protein)_, and G_solv (ligand)_ are the free energies of solvation of protein-ligand complex, protein, and ligand, respectively.

- The surface area energies (ΔG_SA_) could be calculated via equation 4

ΔG_SA_ = _­_ G_SA(complex)_ – (G_SA (protein)_ + G_SA (ligand)_ ----------------Eq.4

G_SA(complex)_, (G_SA (protein)_, and G_SA (ligand)_ are the surface free energies of a protein-ligand complex, protein, and ligand, respectively.

**Figure S10. Crystal binding mode of celecoxib, and predicted binding orientations, visualized in COX-1 active site**

**
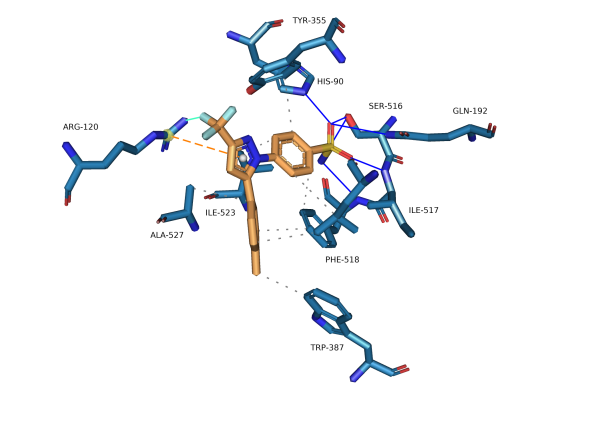
**

**Figure S11. Crystal binding mode of 2d compound, and predicted binding orientations, visualized in COX-1 active site**

**
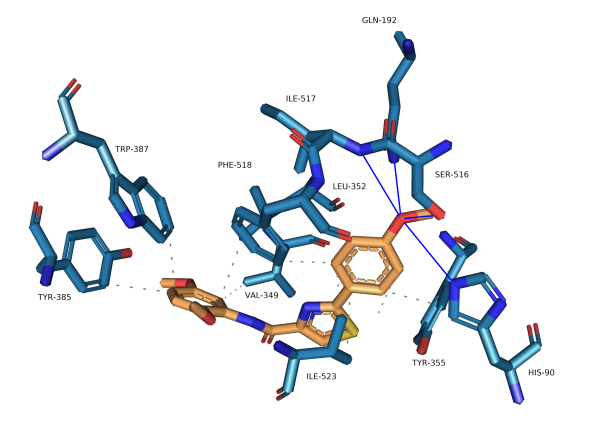
**

**Figure S12. Crystal binding mode of 2e compound, and predicted binding orientations, visualized in COX-1 active site
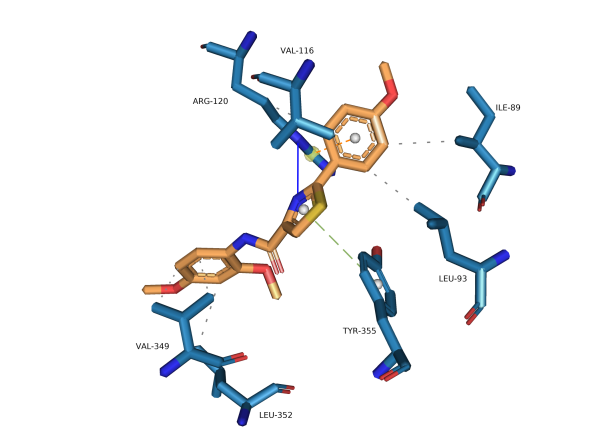
**

**Figure S13. Crystal binding mode of 2f compound, and predicted binding orientations, visualized in COX-1 active site**

**
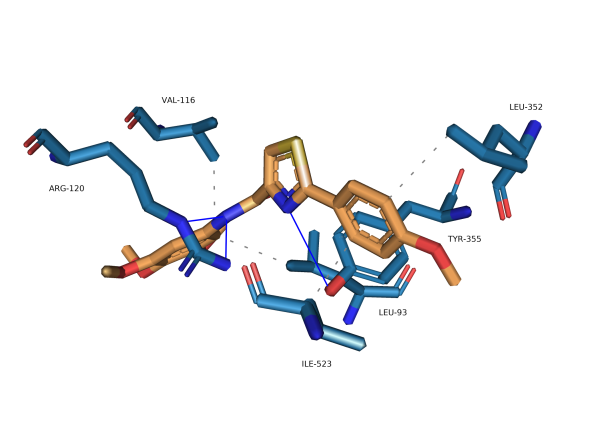
**

**Figure S14. Crystal binding mode of 2i compound, and predicted binding orientations, visualized in COX-1 active site**

**
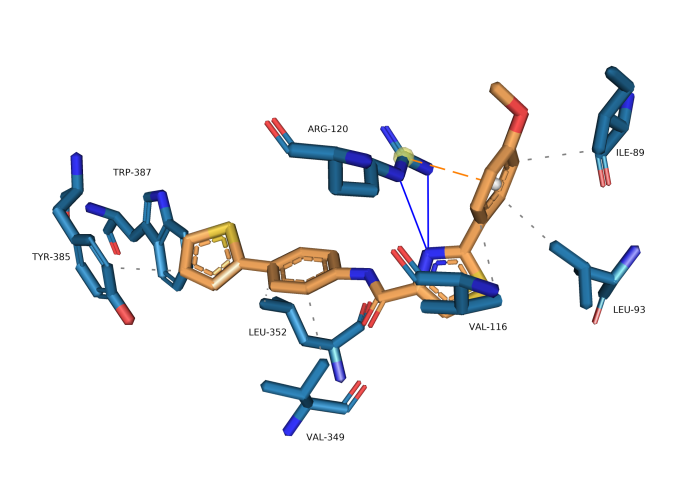
**

**Figure S15. Crystal binding mode of celecoxib, and predicted binding orientations, visualized in COX-2 active site**

**
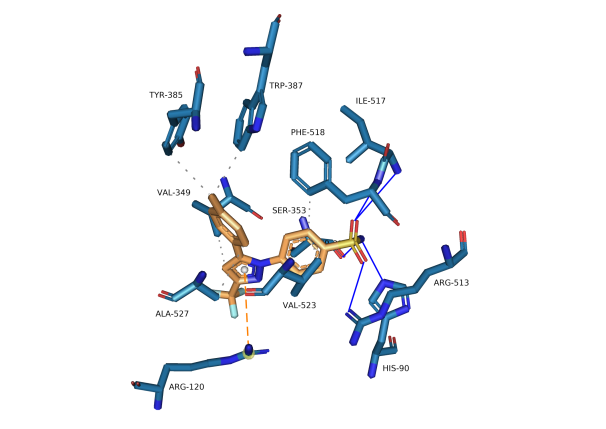
**

**Figure S16. Crystal binding mode of 2d compound, and predicted binding orientations, visualized in COX-2 active site**

**
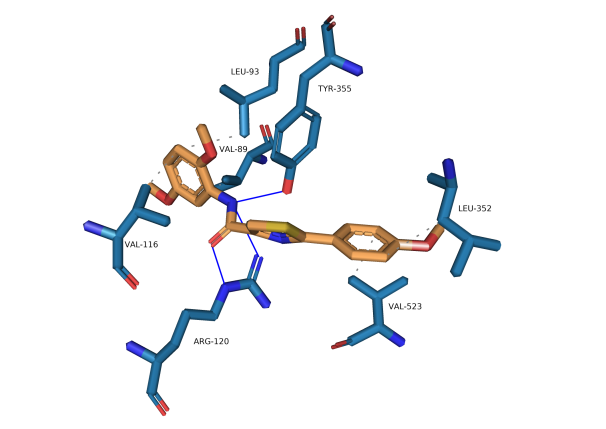
**

**Figure S17. Crystal binding mode of 2e compound, and predicted binding orientations, visualized in COX-2 active site
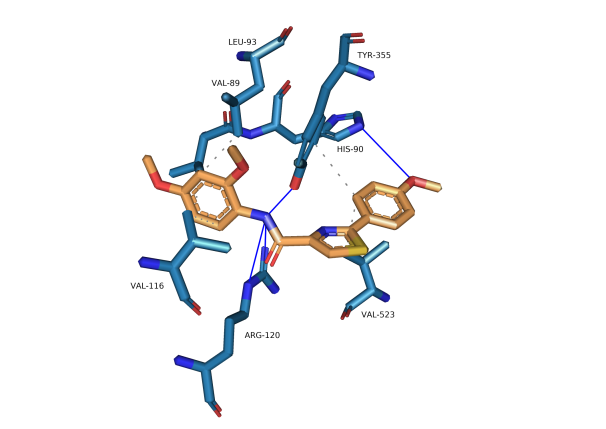
**

**Figure S18. Crystal binding mode of 2f compound, and predicted binding orientations, visualized in COX-2 active site**

**
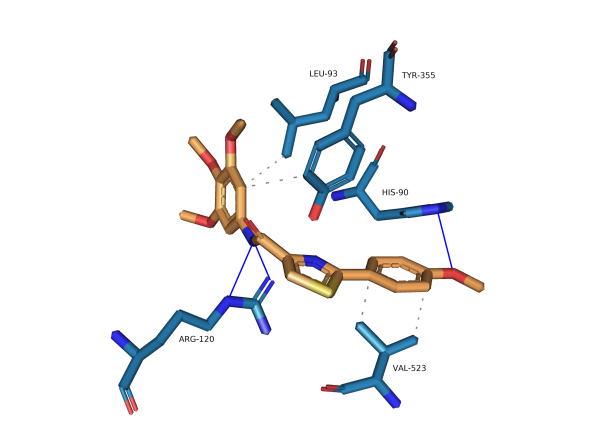
**

**Figure S19. Crystal binding mode of 2i compound, and predicted binding orientations, visualized in COX-2 active site**

**
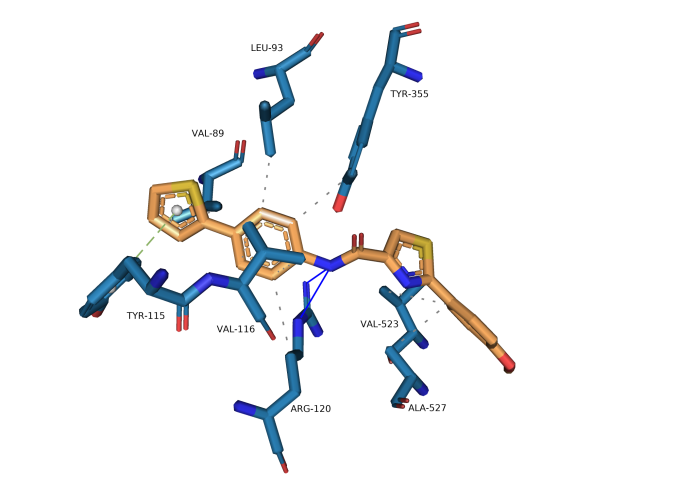
**

**Figure S20:** Plots of highest occupied molecular orbital (HOMO) and lowest unoccupied molecular orbital (LUMO) of compounds **2a**, 2b, 2c, 2g and 2h. The positive electron density has been shown in red color while negative have been shown in blue.

| **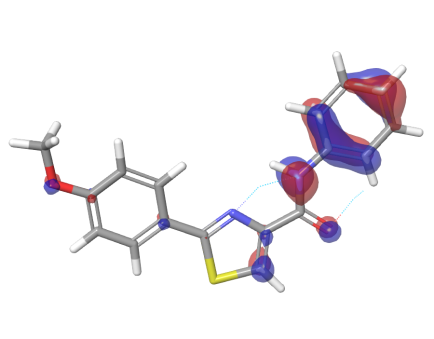**  **2a HOMO** | **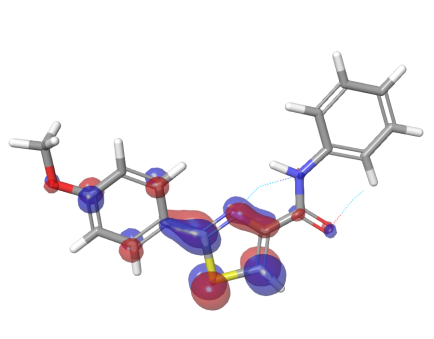**  **2a LUMO** |
| --- | --- |
| **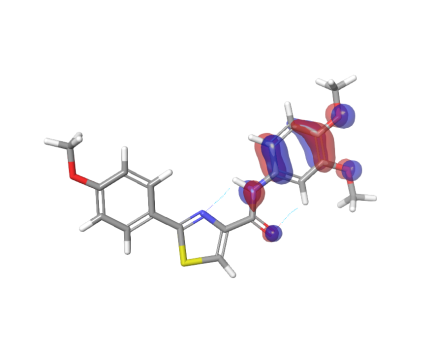**  **2b HOMO** | **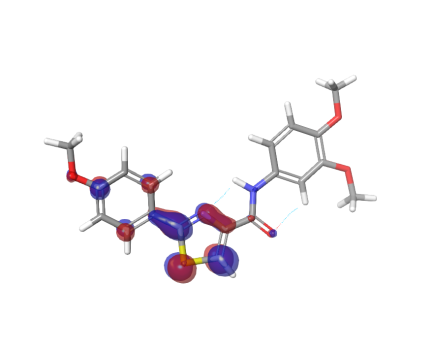**  **2b LUMO** |
| **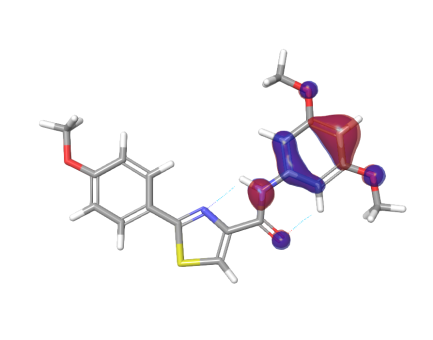**  **2c HOMO** | **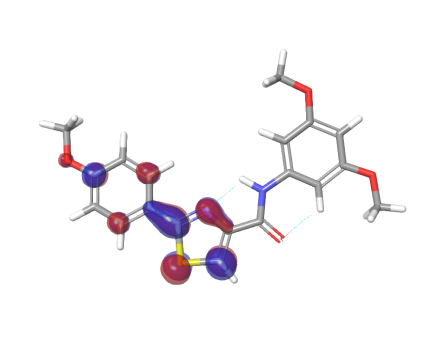**  **2c LUMO** |
| **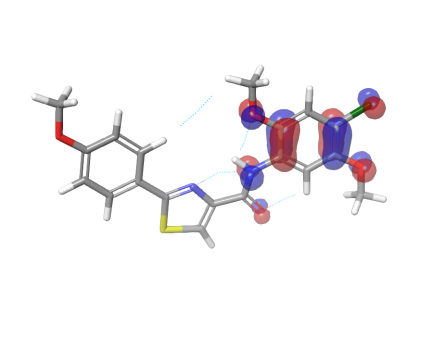**  **2g HOMO** | **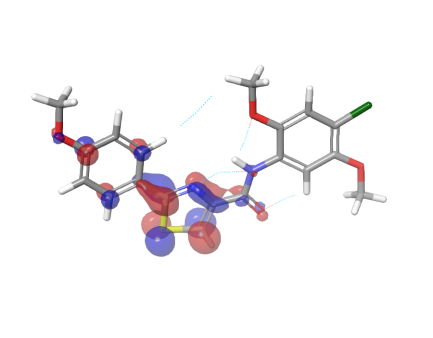**  **2g LUMO** |
| **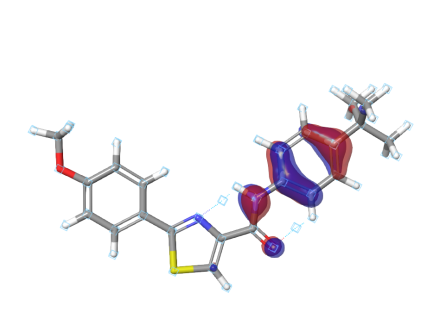**  **2h HOMO** | **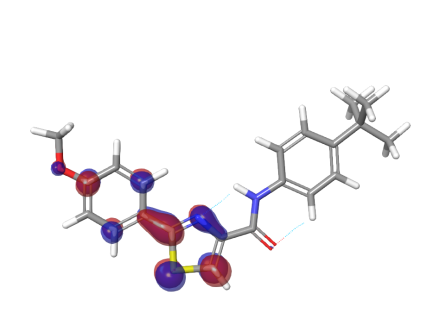**  **2h LUMO** |

**Table S1:** The ADME-T properties of synthesized molecules using QiKProp module (schrödinger 12.1, LLC, NY) running in normal mode. **Mol Mw:** molecular weight of the molecule. **SASA:** total solvent accessible surface area (SASA) in square angstroms using a probe with a 1.4 Å radius. **FOSA:** Hydrophobic component of the SASA (saturated carbon and attached hydrogen). **FISA:** Hydrophilic component of the SASA (SASA on N, O, H on heteroatoms, carbonyl C). **PISA:** π (carbon and attached hydrogen) component of the SASA. **WPSA:** Weakly polar component of the SASA (halogens, P, and S). **Volume:** Total solvent-accessible volume in cubic angstroms using a probe with a 1.4 Å radius. **QPpolrz:** Predicted polarizability in cubic angstroms. **PSA:** van der Waals surface area of polar nitrogen and oxygen atoms and carbonyl carbon atoms. **Percent Human Oral Absorption:** predicted human oral absorption on a 0–100% scale. **Dipole:** computed dipole moment of the molecule. **QPlogPo/w:** predicted octanol/water partition coefficient. **QPlogPoct++:** predicted octanol/gas partition coefficient. **QPlogBB:** predicted brain/blood partition coefficient. **QPlogHERG:** Predicted IC_50_ value for blockage of HERG K+ channels. **QPlogS:** predicted aqueous solubility, S in mol dm^−3^. **QPlogKhsa:** prediction of binding to human serum albumin. **Rule of three:** number of violations of Jorgensen’s rule of 3. **Rule of five:** number of violations of Lipinski rule of 5.

|  | | **Compounds** | | | | | | | | | **Recommended**  **values** |
| --- | --- | --- | --- | --- | --- | --- | --- | --- | --- | --- | --- |
|  |  | **2a** | **2b** | **2c** | **2d** | **2e** | **2f** | **2g** | **2h** | **2i** |  |
| **ADMET parameters** | **Mol_MW** | 310.37 | 370.42 | 370.42 | 370.42 | 370.42 | 400.44 | 404.86 | 366.47 | 392.49 | 130-725 |
|  | **PSA** | 54.07 | 69.74 | 70.45 | 69.05 | 69.06 | 77.24 | 68.31 | 54.09 | 54.10 | 7 – 200 |
|  | **SASA** | 592.7 | 668.1 | 666.9 | 650.5 | 650.8 | 708.4 | 680.0 | 693.5 | 699.5 | 300.0 – 1000.0 |
|  | **FOSA** | 92.93 | 277.7 | 277.0 | 269.6 | 268.7 | 359.5 | 270.1 | 285.2 | 93.30 | 0.0 – 750.0 |
|  | **FISA** | 55.08 | 53.46 | 53.67 | 54.20 | 55.99 | 53.50 | 54.05 | 55.12 | 54.96 | 7.0 – 330.0 |
|  | **PISA** | 402.3 | 294.5 | 293.9 | 283.8 | 283.3 | 252.4 | 242.0 | 310.8 | **467.3** | 0.0 – 450.0 |
|  | **WPSA** | 42.35 | 42.34 | 42.35 | 42.80 | 42.71 | 42.93 | 113.8 | 42.34 | 83.96 | 0.0 – 175.0 |
|  | **Volume** | 994.12 | 1144.7 | 1145.5 | 1143.3 | 1143.4 | 1233.8 | 1190.4 | 1209.3 | 1200.2 | 500.0 – 2000.0 |
|  | **QPpolrz** | 35.54 | 39.26 | 39.28 | 39.10 | 39.10 | 41.78 | 40.59 | 42.64 | 44.41 | 13.0 – 70.0 |
|  | **Percent Human Oral Absorption** | 100 | 100 | 100 | 100 | 100 | 100 | 100 | 100 | 100 | >80% is high  <25% is low |
|  | **Dipole** | 5.99 | 6.85 | 6.89 | 6.03 | 7.75 | 7.36 | 6.82 | 5.85 | 6.83f | 1.0-12.5 |
|  | **QPlogPo/w** | 3.77 | 3.96 | 3.97 | 3.95 | 3.93 | 4.16 | 4.47 | 5.01 | 5.30 | -2.0-6.5 |
|  | **QPlogPoct++** | 16.18 | 18.06 | 18.08 | 17.95 | 18.24 | 19.28 | 18.81 | 18.34 | 19.55 | 8.0-35 |
|  | **QPlogBB** | -0.06 | -0.20 | -0.20 | -0.18 | -0.19 | -0.27 | -0.02 | -0.16 | -0.01 | -3 – 1.2 |
|  | **QPlogHERG** | -6.48 | -6.30 | -6.27 | -5.91 | -5.91 | -6.21 | -5.94 | -6.38 | -7.30 | below  –5 |
|  | **QPlogS** | -5.05 | -5.51 | -5.49 | -5.20 | -5.20 | -5.77 | -6.03 | **-6.68** | **-7.14** | -6.0-0.5 |
|  | **QPlogKhsa** | 0.26 | 0.27 | 0.28 | 0.28 | 0.28 | 0.31 | 0.40 | 0.80 | 0.81 | -1.5 – 1.5 |
|  | **Rule of three** | 0 | 0 | 0 | 0 | 0 | 1 | 1 | 1 | 1 | <3 |
|  | **Rule of five** | 0 | 0 | 0 | 0 | 0 | 0 | 0 | 1 | 1 | <4 |
